# Recent Advances in Chemical Synthesis of Amino Sugars

**DOI:** 10.3390/molecules28124724

**Published:** 2023-06-12

**Authors:** Jian Yang, Demeng Xie, Xiaofeng Ma

**Affiliations:** 1 Chengdu Institute of Biology, Chinese Academy of Sciences, Chengdu 610041, China; yangjian183@mails.ucas.ac.cn (J.Y.); xiedm@cib.ac.cn (D.X.); 2 University of Chinese Academy of Sciences, Beijing 100049, China

**Keywords:** amino sugars, biological activities, stereoselective synthesis, glycosylation

## Abstract

Amino sugars are a kind of carbohydrates with one or more hydroxyl groups replaced by an amino group. They play crucial roles in a broad range of biological activities. Over the past few decades, there have been continuing efforts on the stereoselective glycosylation of amino sugars. However, the introduction of glycoside bearing basic nitrogen is challenging using conventional Lewis acid-promoted pathways owing to competitive coordination of the amine to the Lewis acid promoter. Additionally, diastereomeric mixtures of *O*-glycoside are often produced if aminoglycoside lack a C2 substituent. This review focuses on the updated overview of the way to stereoselective synthesis of 1,2-*cis*-aminoglycoside. The scope, mechanism, and the applications in the synthesis of complex glycoconjugates for the representative methodologies were also included.

## 1. Introduction

**Figure 1:**
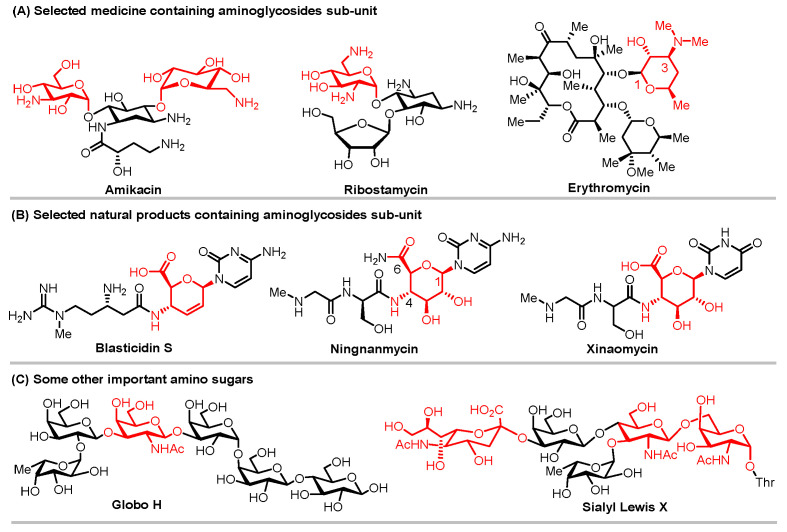
Selected Natural products and pharmaceuticals containing amino sugars. (**A**) Selected medicine containing aminoglycosides sub-unit, (**B**) Selected natural products containing aminoglycosides sub-unit, (**C**) Some other important amino sugars.

A particular kind of carbohydrates known as amino sugars have one or more hydroxyl groups replaced by amino groups. Amino sugars exist ubiquitously in nature and are present in many biologically important and naturally occurring oligosaccharides and natural products. These molecules are closely related to people’s lives and play a vital role in the human battle against diseases, and increase the quality and the yield of grain. For instance, erythromycin and other aminoglycoside and macrolide antibiotics, including amikacin and ribostamycin, which all incorporate the amino sugar sub-unit and are used as the first-line therapeutic medications for the treatment of bacterial infections, have prevented millions of deaths (Figure 1A). Amino sugars are also found in a wide range of natural products, such as Blasticidin S, Ningnanmycin, and Xinaomycin, and have been utilized successfully as agricultural antibiotics [1,2,3] (Figure 1B). Additionally, sialic acids, which are essential for cell mutual recognition, differentiation, adhesion, and malignancy, and 2-amino sugars, which are widely prevalent in various sugar complexes like polysaccharides, glycopeptides, glycolipids, and glycoproteins are both unique amino sugars. (Figure 1C) [4]. At the present stage, the availability of such compounds relies heavily on microbial fermentation, however, it is hard to quickly construct compound libraries containing multiple structural analogues in efficient ways. As a result, they are not suitable for a broad range of bioactivity screening and structure–activity relationship studies, which, ultimately, restricts the development of their activity and exploration of their functionality. 

On the other hand, chemical synthesis of aminoglycosides is always associated with great challenge because: (i) the inherent regioselectivity and stereoselectivity issues in chemical synthesis of carbohydrates resulted in the lack of a general protocol to construct glycosides in an efficient manner [5,6]; (ii) competitive coordination of the basic amine to the Lewis acid promoter renders the traditional glycosylation conditions inefficient [7,8,9,10,11]; and (iii) many amino monosaccharides are not commercially available or available at a high price and often require the introduction of amino groups into the scaffolds by chemical methods through multi-step protecting/deprotecting procedures, which still encountered regio- and stereo-selective issues [12].

Despite these challenges, in view of the structural and pharmacological diversity of aminoglycosides, many elegant chemical methods were thus developed for the synthesis of aminoglycosides. In this review, we wish to give the reader an overview of the recent achievements in stereoselective synthesis of aminoglycosides by using amino sugars as glycosyl donors. Since 6-amino-6-deoxy sugars could be readily obtained from corresponding 6-OH analogues, and there are several graceful articles and reviews on the synthesis of 1-amino-1-deoxy sugars (*N*-glycosides) [13,14], this review does not include the synthesis of 6-amino-6-deoxy sugars and 1-amino-1-deoxy sugars, but focuses on the chemical synthesis of 2-amino-2-deoxy, 3-amino-3-deoxy, and 4-amino-4-deoxy glycosides. This mini-review is divided into the following parts: (1) glycosylation of 2-amino-2-deoxysugars with different glycosyl donors; and (2) glycosylation of 3-amino-3-deoxysugars and 4-amino-4-deoxysugars.

## 2. Glycosylation of 2-Amino-2-Deoxysugars

**Figure 2:**
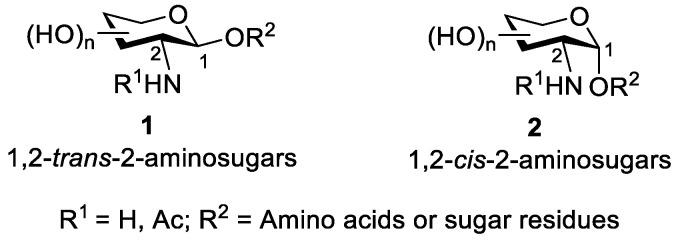
Structure of 1,2-*cis*- and 1,2-*trans*-aminosugars.

2-Amino-2-deoxysugars are commonly distributed in a wide variety of naturally occurring oligosaccharides and glycoconjugates, where they are connected to other residues via anomeric carbon to form 1,2-*cis*-2-aminosugars (*a*-anomer) or 1, 2-*trans*-2-aminosugars (*β*-anomer) [15,16] (Figure 2). In interactions between cells, many of these 2-aminosugars on cell surfaces serve as ligands for proteins, including enzymes, antibodies, and lectins [17,18,19]. With the increasing awareness of the biological importance of 2-amino-2-deoxysugars, many efficient regio- and stereo-selectively chemical methods for the synthesis of oligosaccharides containing these residues have been developed.

**Scheme 1:**
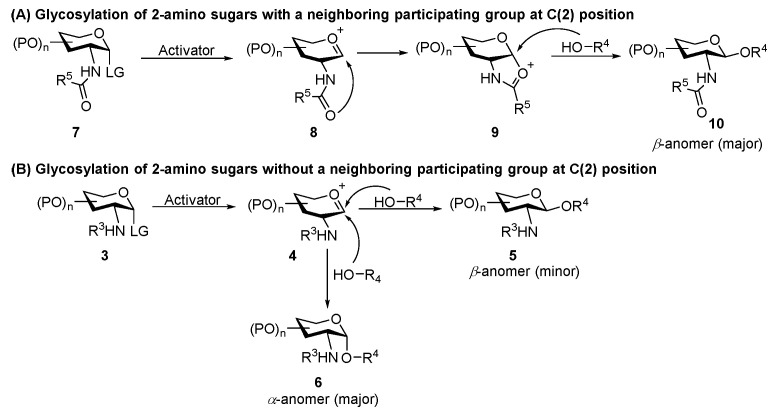
The strategy for the formation of glycoside of 2-amino sugars (**A**) Glycosylation of 2-amino sugars with a neighboring participating group at C(2) position, (**B**) Glycosylation of 2-amino sugars without a neighboring participating group at C(2) position.

**Scheme 2:**
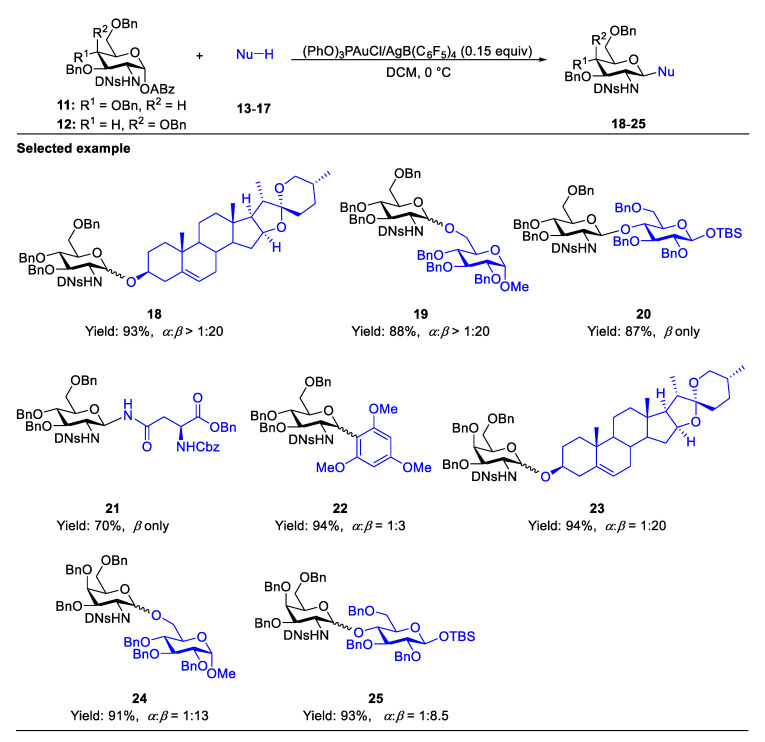
Glycosylation of DNs-protected donor **11** and **12** with various nucleophiles.

**Figure 3:**
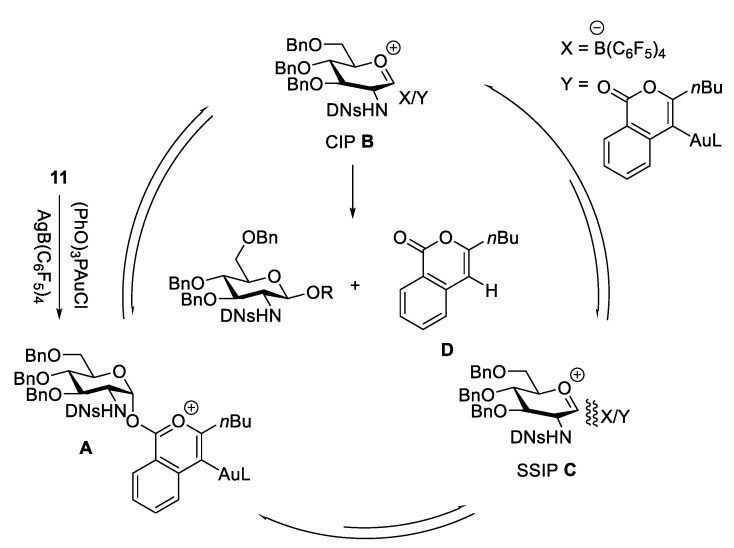
Proposed mechanism for glycosylation of **11**.

**Scheme 3:**
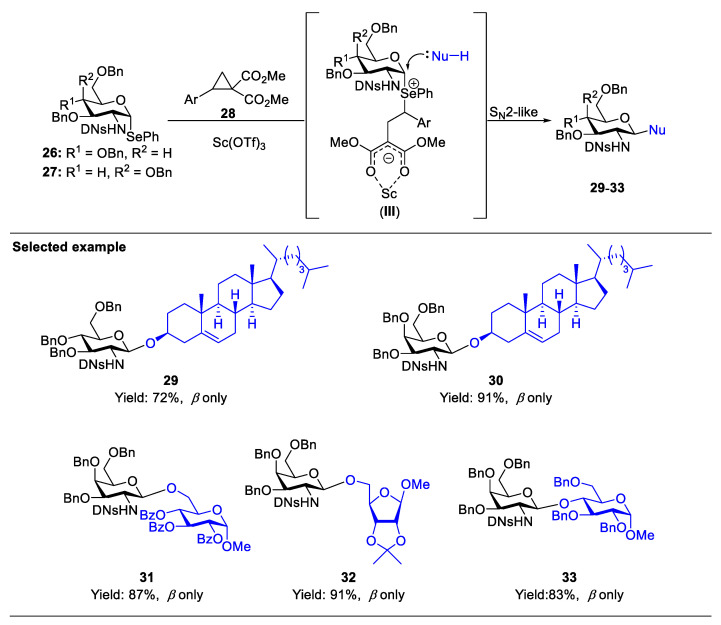
DNs-directed S_N_2-like glycosylation of selenoglycosaminosides.

The formation of 1,2-*trans*-aminoglycoside which exists in nature such as chitin, chitosan, as well as many *O*-linked GlcNAc targets and *N*-linked glycans, can be reliably performed with the assistance of a neighboring participating group at C(2)-amide to provide the *β*-linked product **10** (Scheme 1A) [20,21,22]. There are several elegant reviews on the stereoselective formation of 1,2-*trans*-2-aminoglycoside [16,23,24,25]. Here, we only deal with two of the very recent representative examples. As for a non-participatory group at C(2)-amide, Li’s group developed an effective strategy for *β*-selective glycosylation of 2,4-dinitrobenzenesulfonyl (DNs)-protected glucosaminosyl and galactosaminosyl donors using Yu’s glycosyl *o*-hexynylbenzoates as the donor (Scheme 2) [26]. The methodology is compatible with a wide range of substrates, including *O*-, *N*-, and *C*-nucleophiles. A plausible mechanism suggested that the reaction was achieved through a S_N_2-like pathway. It was supposed that, compared to solvent-separated ion pair (SSIP) intermediate **C**, the equilibrium heavily shifts toward the intermediates glycosyloxyisochromenylium **A** and contact ion pair (CIP) **B** due to the instability of **C** caused by the strong electron-withdrawing effect of C(2)-DNsNH. Moreover, nucleophiles preferentially attack intermediates **A** and **B** to stereocontrollably result in *β*-glucosaminosides in an S_N_2-type process (Figure 3). Very recently, Liu and co-workers developed a donor-acceptor cyclopropane (DAC)/Sc(OTf)_3_ system to activate C(2)-DNsNH-protected selenoglycoside donors (Scheme 3) [27]. The mechanism suggested the DNs group would stabilize the glycosyl zwitterion intermediate (**III**) to engage in the S_N_2-like displacement to form the 1,2-*trans* glycosaminyl bonds in a nonparticipating manner.

1,2-*cis*-Aminoglycoside, the key constituents in many naturally occurring bioactive glycoconjugates, are found to have a wide range of biological activities [15,16], therefore, it is meaningful to study the corresponding synthetic methodologies. The preparation of 1,2-*cis*-aminoglycoside **6** is challenging as the most readily amide protecting group for amine would produce the undesired 1,2-*trans*-product **5** due to the participation of the neighboring-group (Scheme 1A), while a C(2)-non-participatory group would unpredictably lead to the anomeric mixtures at the newly formed glycosidic bond, even though the 1,2-*cis*-anomer **6** would be produced as the major product due to thermodynamic stability (Scheme 1B). Herein, we will discuss the methodologies for the construction of 1,2-*cis*-aminoglycoside, according to the employed glycosyl donors. The associated mechanism, the influence of additive to the stereoselectivity, and the application of the methodology to the synthesis of some biologically important oligosaccharides and glycoconjugates will also be included.

### 2.1. Glycosylation with C(2)-Azido Donors

The C(2)-azido functionality serves as an excellent latent amine-precursor that is stable in both acidic and basic circumstances, and can, subsequently, be selectively converted into the amino group, giving the most versatile route for the formation of 1,2-*cis*-aminoglycoside. Due to the chemical compatibility of C(2)-azido with many different kinds of glycosyl donors, including glycosyl halides, trichloroacetimidates, and thioglycosides, it has been applied in total syntheses of a broad range of biologically relevant oligosaccharides and glycoconjugates [28]. The needed C(2)-azide glycosyl donors could be easily prepared from the corresponding 2-amino sugars or by azidation of 2-*O*-triflate. The radical process from glycal was also developed by Tingoli and co-workers by using PhI(OAc)_2_ as radical initial and NaN_3_ as N_3_ source [29]. All of these protocols supported the application of C(2)-azido glycosyl donor for glycosylation.

**Scheme 4:**
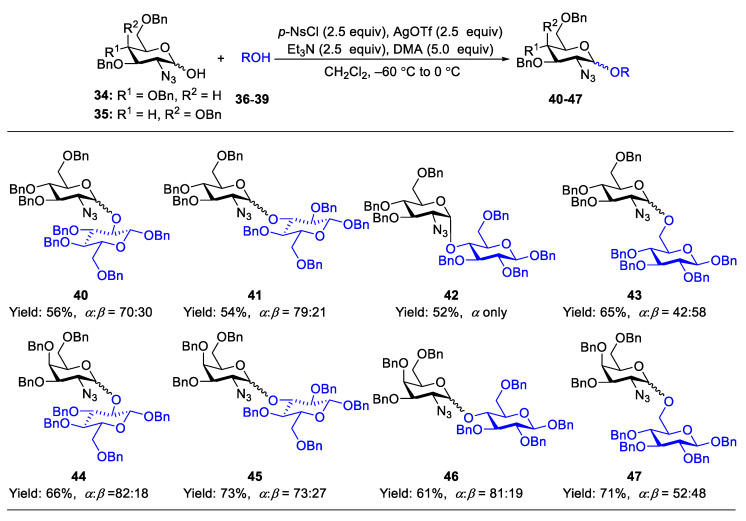
Synthesis of *α*-disaccharides by in situ activation of anomeric OH in **34** and **35**.

Glycosylation by in situ activation of 1-OH sugar derivatives is convenient because it avoids preparation of reactive glycosyl donors. Koto et al. reported the synthesis of *α*-/(1,2-*cis*)-glycosylation of 2-azido-3,4,6-tri-*O*-benzyl-2-deoxy-D-glucose and 2-azido-3,4,6-tri-*O*-benzyl-2-deoxy-D-galactose, which were prepared from corresponding glycals via azido-nitration followed by hydrolyzing 1-*O*-nitrated product on silica-gel column. By employing *p*-nitrobenzenesulfonyl chloride (NsCl)-silver triflate-triethylamine and *N*,*N*-dimethylacetamide (DMA) as the activating system, the anomeric free hydroxyl group could be activated in situ to produce desired products in moderate-to-good yields. The addition of DMA is crucial to control the stereoselectivity as the similar reaction in the absence of DMA lead to the formation of 1,2-*trans*-isomers (Scheme 4) [30].

**Scheme 5:**
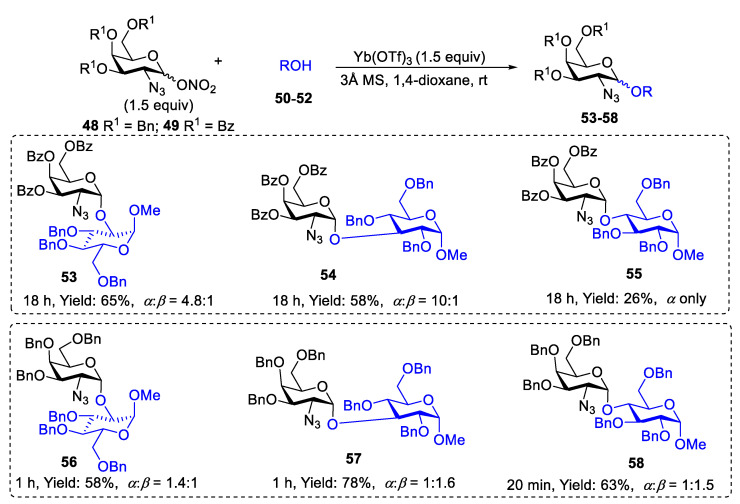
Glycosylation with benzoylated and benzylated galactosyl nitrate.

**Scheme 6:**
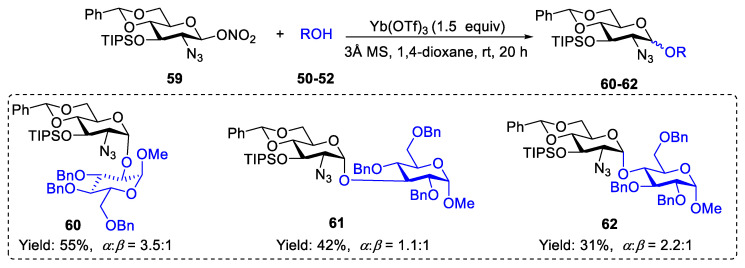
Glycosylation with 2-azidoglucosyl nitrate donor **59**.

Glycosyl nitrates are important synthetic intermediates which could be applied in subsequent functional group transformations. Demchenko’s group reported the utility of glycosyl nitrates as donors for *O*-glycosidation of 2-amino-2-deoxysugars in the presence of ytterbium(III) trifluoromethanesulfonate (Yb(OTf)_3_) (Scheme 5 and Scheme 6) [31]. Coupling the benzoylated donor **48** with acceptors **50**–**52** generates the desired disaccharides in 26–65% yields with good to excellent stereoselectivity (Scheme 5). In contrast to the Bz-protecting group, however, Bn-protected glycosyl nitrates delivered the corresponding products in shorter reaction time, higher yield, but worse stereoselectivity. The author concluded that this is because of the inverse correlation between the reactivity and stereoselectivity. Besides, conformation-restrainin sugar such as 2-azido-4,6-*O*-benzylidene-2-deoxy-3-*O*-triisopropylsilyl-*β*-D-glucosyl nitrate donor **59**, was subjected to the standard reaction condition to couple with glycosyl acceptors **50**–**52**. Despite the fact that the conformation-restraining 4,6-*O*-benzylidene-protected glucosyl donor produces the glycosylated product with *α*-selectivity [32,33,34,35], this reaction merely produced the products with lower yield and inferior selectivity (Scheme 6).

**Scheme 7:**
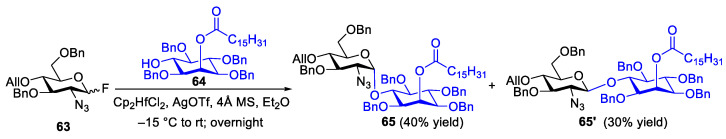
The synthesis of glycosylphosphatidylinositol (GPI) core by Guo’s group.

During synthesis of the core structures of glycosyl phosphatidylinositol (GPI), Guo’s group used the 2-azido-2-deoxy-D-glucopyranose donor **63** to couple with inositol acceptor **64**, generating the desired product **65** and its *β*-isomer **65**’ in 70% yield with 4:3 *α*-/*β*-selectivity (Scheme 7) [36]. The two isomers can be separated by silica gel column, facilitating its further application. Even though no detailed experimental procedure was disclosed, they used dichlorohafnocene (Cp_2_HfCl_2_) and AgOTf as promotors, which may limit its application in large-scale reactions.

**Scheme 8:**
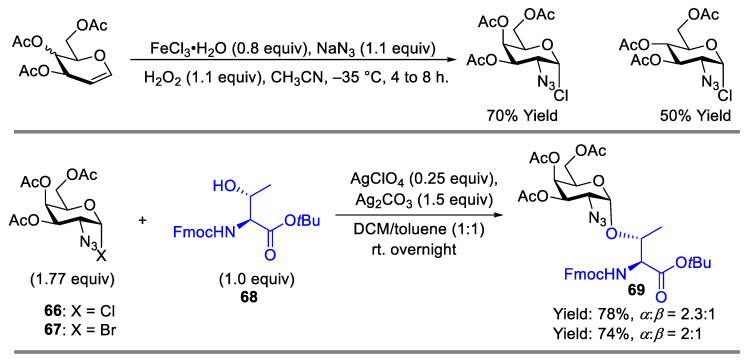
The synthesis of the T_N_-antigen building block.

In 2011, Sewald’s group reported a one-pot procedure to prepare 2-azido glycosyl chlorides via FeCl_3_/H_2_O_2_ mediate azidochlorination of glycals. A series of mono- and disaccharide 2-azido glycosyl chlorides were synthesized from corresponding glycals in 37–78% yields with high *α*-selectivity. The reaction could also be carried out at 15 g scale which produced the product in similar yield and stereoselectivity. The produced 2-azidated galactosyl donor **66** was then used to prepare glycosylated threonine building block **69** under Koenigs–Knorr glycosylation conditions. The glycosylated amino acid derivative **69** could be used as the substrate for the synthesis of T_N_-antigen (*α*-GalNAc-Ser/Thr) (Scheme 8) [37]. The yields and diastereoselectivities of the threonine glycosylation are also comparable to the corresponding bromide **67**, which was obtained from an azidonitration reaction followed by bromination [38,39].

**Scheme 9:**
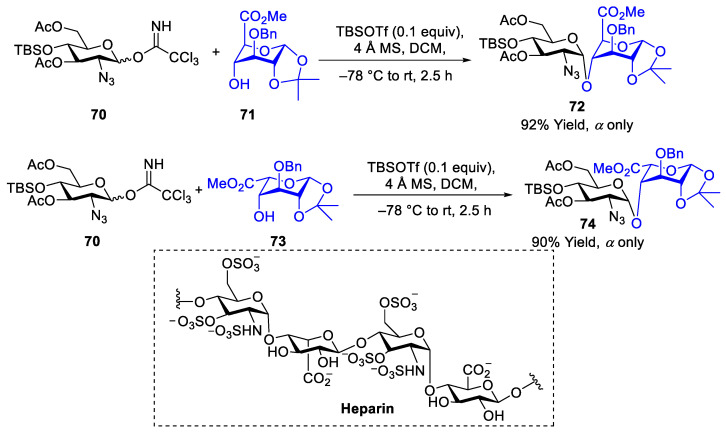
The construction of a 2-amino-*α*-glycosidic bond by using conformation-locked acceptors.

Heparin, a kind of glycosaminoglycan, is crucial to a number of important biological functions [40]. Highly selective installation of the 2-amino-*α*-glycosidic linkage in the heparin backbone is challenging [41]. In 2002, Seeberger’s group reported a new method for the stereoselective installation of *α*-glucosamine linkages by locking the conformation of the monosaccharide acceptors (Scheme 9) [42]. Coupling the donor **70** with conformation-locked glucuronic acid acceptor **71** and iduronic acid acceptor **73** provides the desired disaccharides **72** and **74** in 92% and 90%, respectively, with completely *α*-selectivity.

**Scheme 10:**
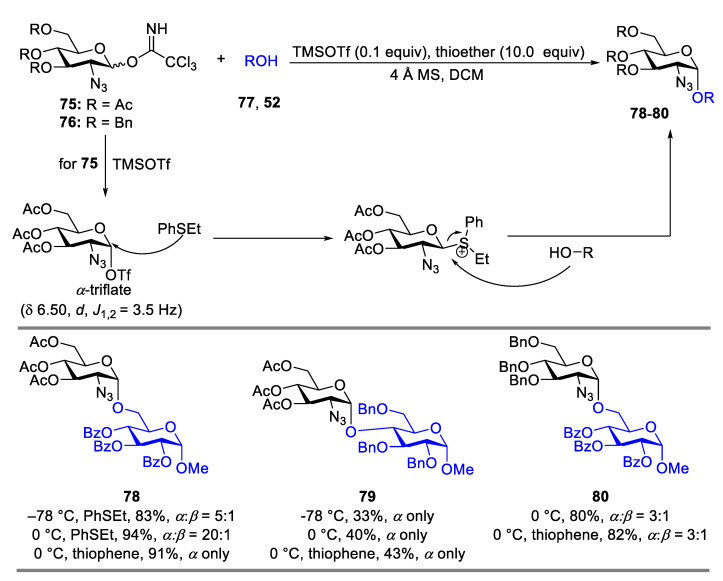
The glycosylation of glycosyl donors **75** and **76** with glycosyl acceptors **77** and **52** in the presence of thiophene.

In 2007, Geert-Jan Boons and co-workers reported the glycosylation of 2-azido-2-deoxy-glycosyl trichloroacetimidates **75** and **76** with a variety of glycosyl acceptors in the presence of PhSEt or thiophene to provide disaccharides in good-to-high yields with excellent *α*-selectivity (Scheme 10) [43]. The enhanced *α*-selectivity by addition thioether was proved to be derived from successive configurational inversion via S_N_2-type reactions, that is, the thioether reacted with *α*-triflate which was confirmed by chemical shift and a smaller coupling constant, producing the *β*-substituted anomeric sulfonium ion then glycosyl acceptors attacked the anomeric sulfonium ion to generate the product with high *α*-selectivity. It was also found that in most cases: (i) elevating the reaction temperature could dramatically improve the stereoselectivity; and (ii) thiophene produced the products with better *α*-selectivity compared with ethyl phenyl sulfide (PhSEt). However, a similar reaction with acceptor **52** produced the product **79** in lower yield with only *α*-selectivity regardless of an increase in the reaction temperature or in the presence of thioether; the lower yield was because of the formation of an anomeric trichloroacetamide by-product. They also synthesized the 3,4,6-tri-*O*-benzyl-glucopyranosyl donor **76**, and carried out glycosylation reaction under the same conditions with or without thiophene. Similar to the benzyl-protected glycosyl nitrate donors, benzyl-protected glycosyl trichloroacetimidate **76** also delivered the corresponding glycoside in lower stereoselectivity. The author speculated that the glycosylation may take place directly between acceptors with the oxacarbenium ion intermediate produced from the high reactivity of the benzyl-protected glycosyl donor.

**Scheme 11:**
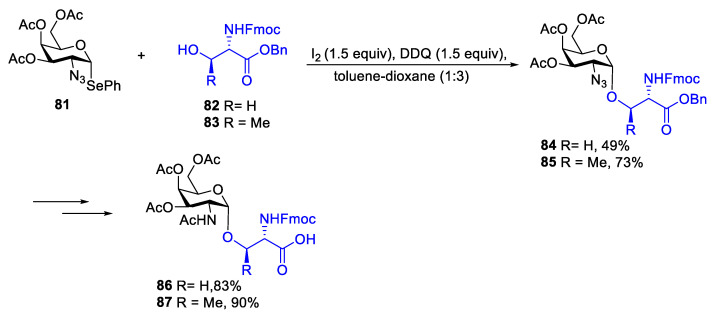
The formation of T_N_-antigen building blocks.

In 2008, Field’s group disclosed synthesis of mucin-related glyco-aminoacids and glycopeptides by using phenylseleno 3,4,6-*tri*-*O*-acetyl-2-azido-2-deoxy-*α*-D-galactopyranoside **81** as glycosyl donor under the promotion of I_2_ and DDQ. In this case, selenogalactoside **81**, which could be readily prepared from galactal via azidophenylselenation, was reacted with serine **82** and threonine **83** derivatives in toluene/dioxane to achieve exclusive *α*-stereoselectivity (Scheme 11) [44]. It was found that the use of toluene/dioxane mixture solvent and DDQ are critical for improving the yield and stereoselectivity. Even though the exact mechanism for DDQ-promoted glycosylation was not clear, they believed that high *α*-stereoselectivity was perhaps due to the formation of the *O*-*β*-glycosyl oxonium ion derived from dioxane.

**Scheme 12:**
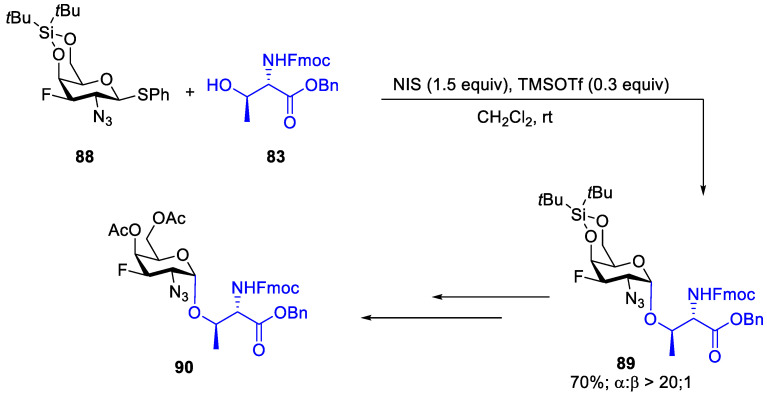
The synthesis and protecting group manipulation of 3-fluoro T_N_ antigen analogues.

Very recently, during the synthesis of deoxyfluorinated T_N_ antigen analogues, Karban and co-workers prepared a series of C3 and C4 deoxyfluorinated galactosazide thiodonors to react with a variety of glycosyl acceptors and threonine derivatives in order to evaluate their stereoselectivity. It was found that for the threonine derivatives, the *O*-benzylated C4 fluoro-donor gave the coupling products in 63% to 69% yield with *α*:*β* selectivity ranged from 2.5:1 to 3.1:1, regardless of solvent (ether solvents such as diethyl ether and dioxane gave similar stereoselectivity as DCM alone). For C3 fluoro-donors, even though the donors with acyl and silyl protection at the 4- and 6-positions did not improve the *α*-selectivity, the reaction between 4,6-di-*tert*-butylsilylene-protected 3-fluoro galactosazide thiodonor **88** and threonine benzyl ester **83** produced the glycoside product **89** in excellent 1,2-*cis*-stereoselectivity (Scheme 12) [45].

**Scheme 13:**
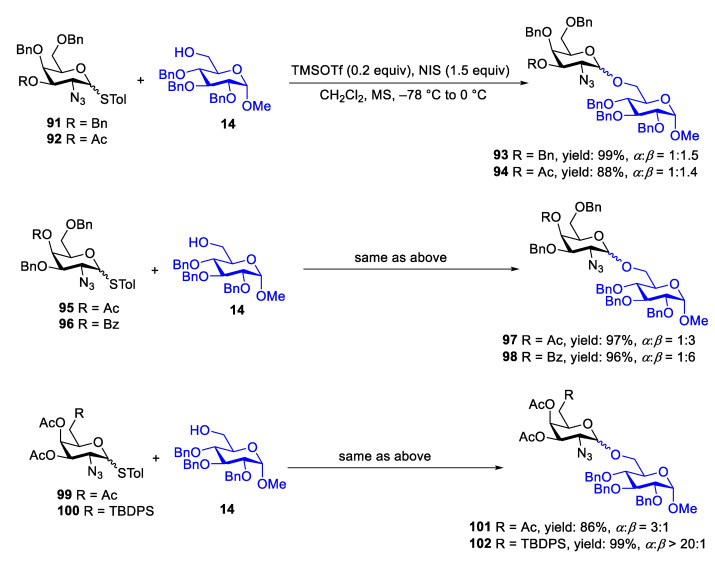
The results of glycosylation.

Due to the protecting groups’ significant impact on the stereoselectivity of glycosyl donors, Li’s group was committed to examining the stereochemistry effect of the protecting groups [46,47,48]. They synthesized a panel of GalN_3_ thioglycoside donors with different protecting groups at 3-, 4-, and 6-positions, and compared the stereoselectivity of the glycosylation with primary alcohol **14**. They discovered that the protecting group at 3-position had no noticeable effect on the stereoselectivity because both benzyl- and acetyl-protected thioglycoside donors delivered the products in comparable selectivity, whereas the acyl-protecting group at 4-position could increase the stereoselectivity with 4-benzoyl-protected thioglycoside producing the product in 96% yield with 1:6 of *α*:*β*. Furthermore, 2-azido-3,4,6-*tri*-*O*-acetyl-1-thio-glycoside produced the glycosylation product in a 3:1 *α*:*β* selectivity, while coupling the 6-TBDPS-protected similar donor **100** with the acceptor **14** gave the desired disaccharide product **102** in 99% yield with *α*:*β* > 20:1 [49] (Scheme 13). They also applied the optimized acyl-thioglycoside with the TBDPS protecting group at 6-position to the synthesis of precursors of T_N_ antigen.

**Scheme 14:**
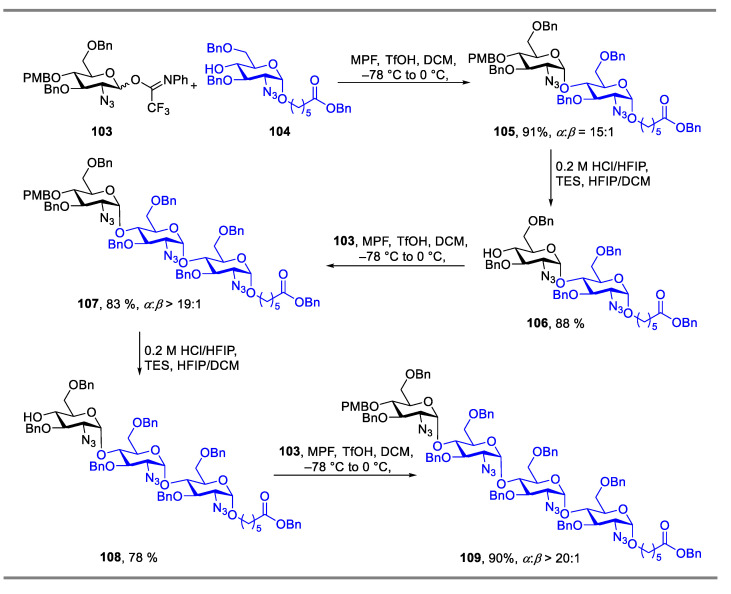
The assembly of an *α*-glucosazide tetrasaccharide using MPF-mediated glycosylation.

**Scheme 15:**
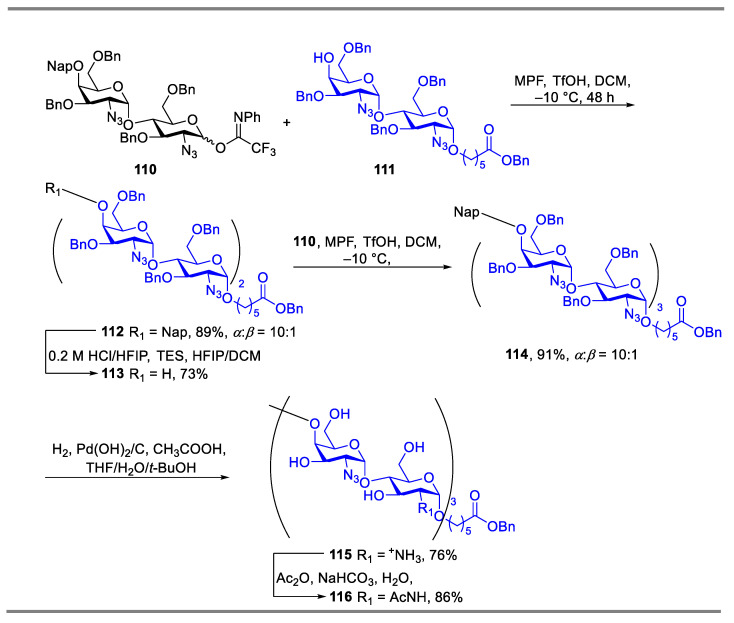
The assembly of a Pel fragment **116**.

The strategy of reagent-controlled glycosylation was also employed in the synthesis of oligosaccharides. Codée’s group reported methyl(phenyl)formamide (MPF) could be employed as an additive for the glycosylation of 2-azido-2-deoxyglucose building blocks [50]. By using the strategy of MPF mediated glycosylation, an all-1,2-*cis*-linked tetraglucosamine was successfully synthetized. The repetitive coupling step of glycosylation reactions mediated by MPF between donor **103** and acceptor under the above identified reaction conditions provided the desired disaccharide in good yield with excellent *α*-selectivity (Scheme 14). Furthermore, using the same method, a fragment of Pel hexasaccharide **116** that contained both GalN and GlcN residues was also effectively synthesized in 86% yield with *α*:*β* = 10:1 (Scheme 15).

### 2.2. Glycosylation with 2-Nitroglycals

**Scheme 16:**
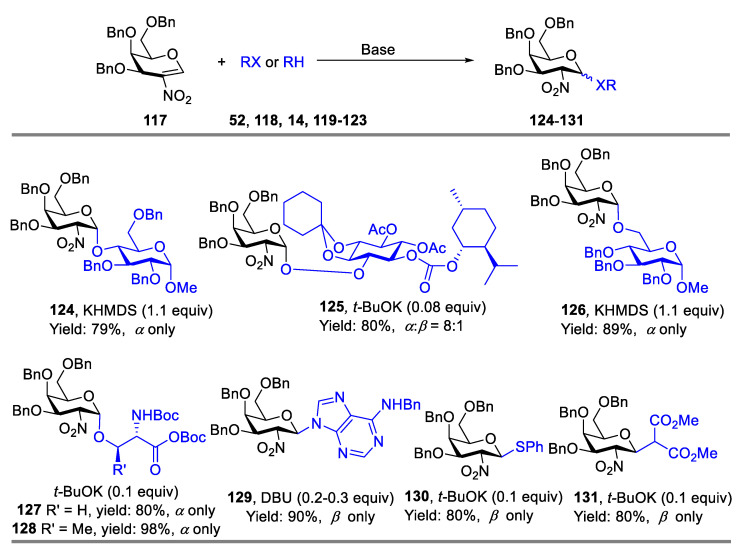
Michael-type addition of alcohols to 2-nitro-D-galactal derivatives.

2-Nitroglycals, which could be readily prepared from corresponding glycals under nitration conditions, are important synthons in carbohydrate chemistry, and are amenable to Michael-type addition under a variety of conditions to produce 2-deoxy-2-nitroglycoside. The produced 2-deoxy-2-nitroglycoside could be readily converted into 2-amino-2-deoxyglycoside under reduction conditions. Since early work by Lemieux and co-workers [51] in the field of glycals, chemical methodologies for stereo- and regio-selective glycosylation with 2-nitroglycals have been broadly developed. Schmidt’s group has extensively studied base-catalyzed glycosylation of benzyl-protected 2-nitrogalactals with various acceptors to construct *O*-/*S*-/*N*-glycoside [52,53,54,55,56,57]. For instance, fully *O*-benzyl-protected 2-nitro-D-galactal **117** was employed by them to couple with monosaccharides, serine or threonine derivatives catalyzed by strong bases such as *t*-BuOK or KHMDS, providing galactosides (**124**–**128**) in high yields with good-to-excellent *α*-stereoselectivity, meanwhile, the same efficient glycosylation was also achieved when *N*,*S*,*C*-nucleophiles were used, and the corresponding *N*-/*S*-/*C*-glycosylation products (**129**–**131**) were produced in very good yield but with *β*-stereoselectivity (Scheme 16) [53,54].

**Scheme 17:**
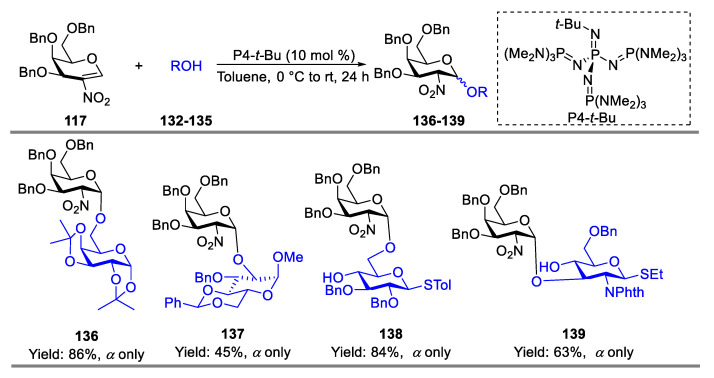
Stereoselective glycosylation of **117** using P4-*t*-Bu as superbase catalyst.

Another example developed by Liu’s group using P4-*t*-Bu as superbase catalyst [58,59], to catalyze stereo- and regioselective glycosylation with 2-nitroglycals [60]. 2-Nitrogalactal underwent *O*-glycosylation with various monohydric alcohol (**132** and **133**) and diol (**134** and **135**) acceptors, providing synthetically useful 2-deoxy-2-nitro-*O*-glycosides in good-to-excellent yields with exclusive *α*-selectivity, while corresponding 2-nitroglucal gave the glycosylation products in high yield but with *β*:*α* ratio ranged from 2:1 to 1:0 (Scheme 17). The author proposed that P4-*t*Bu reacted with alcohol (ROH) to form an ion pair (P4-*t*-BuH^+^ RO^−^) which facilitated alkoxide (RO^−^) addition to 2-nitroglycals. The stereoselectivity of the reaction is presumed because of the steric repulsion between the C4 benzyl group and the alkyl groups on ion pair (P4-*t*-BuH^+^ RO^−^), which repelled alkoxide-attacking 2-nitroglycals from *β*-face for 2-nitrogalactals and *α*-face for 2-nitroglucal, leading to the formation of *α*- and *β*-glycosylation products, respectively.

**Scheme 18:**
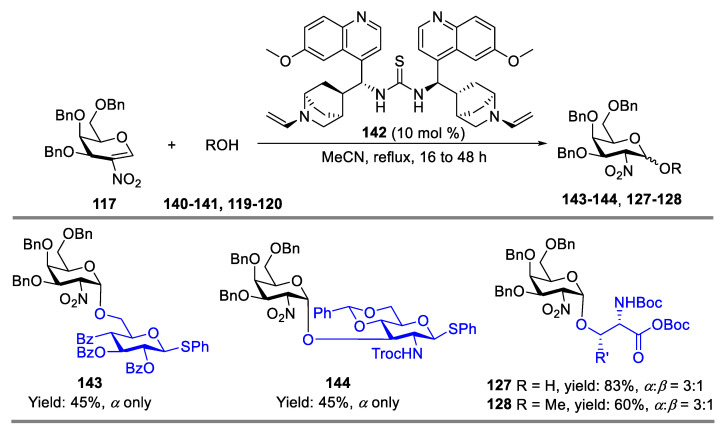
Cinchona-thiourea-catalyzed glycosylation of 2-nitrogalactals **117**.

Due to the excellent H-bond acceptor property of nitro-group, modification of the Schmidt glycosylation protocol to construct 1,2-*cis*-2-aminoglycosidic linkages from 2-nitroglycal by bifunctional organocatalysts and *N*-heterocyclic carbene have been developed in recent years [61,62,63]. Galan’s group reported the first application of a bifunctional cinchona/thiourea organocatalyst **142** for the direct and stereoselective glycosylation of 2-nitrogalactals, affording 2-amino-2-deoxygalactosides in moderate-to-excellent yields with *α*-selectivity [61]. Glycosylation of **116** with glycosyl acceptors bearing electron-withdrawing protecting groups (**140** and **141**) generated disaccharides (**143** and **144)** with complete *α*-stereoselectivity, albeit in moderate yields (45%). Furthermore, serine **119** and threonine derivatives **120** proceeded smoothly to give the corresponding products **127** and **128** in 83% and 60% yield, respectively, and both donors’ stereoselectivity is *α*:*β* = 3:1 (Scheme 18).

**Scheme 19:**
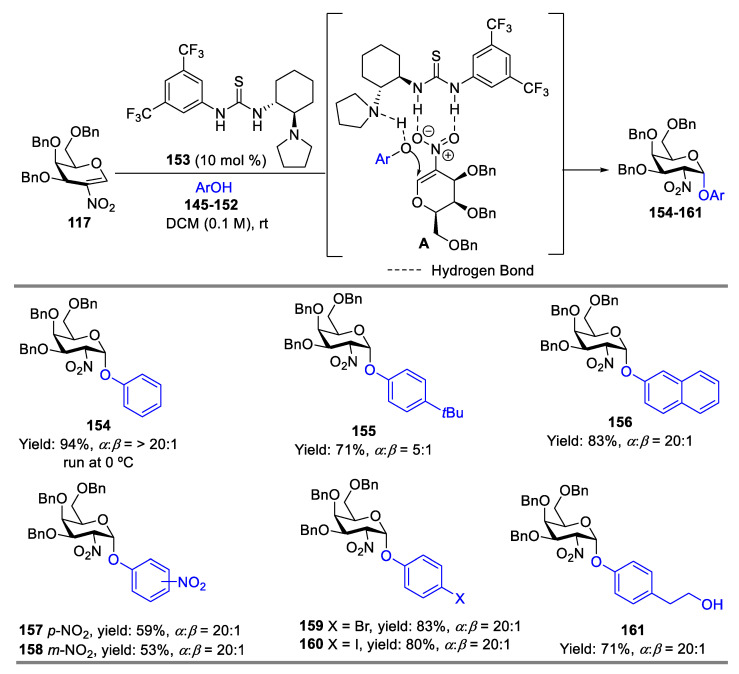
*α*-Selective glycosylation using 2-nitrogalactal **117**.

**Scheme 20:**
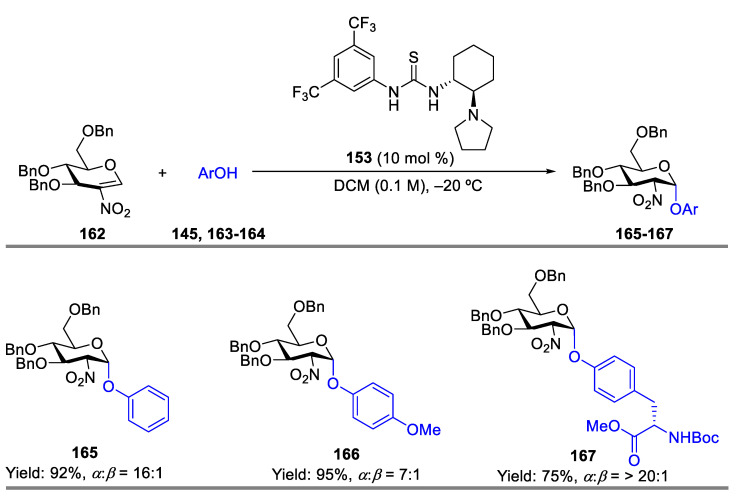
*α*-Selective glycosylation with 2-nitroglucal **162**.

Meanwhile, Takao’s group reported an *α*-selective *O*-glycosylation procedure between 2-nitroglycals and phenol derivatives catalyzed by a bifunctional chiral thiourea catalyst **153** [63]. When 2-nitrogalactal **117** was coupled with acceptors **145**–**152** by thiourea catalyst at room temperature, the desired products were obtained in 53–74% yield with *α*:*β* selectivity ranged from 5:1 to 20:1 (Scheme 19). It was proved that the phenolic hydroxy groups were preferentially glycosylated even in the presence of primary alcohol to provide aromatic-*O*-glycosylation **161**. This could be due to the basic pyrrolidine of the catalyst that assisted in the enhancement of the nucleophilic character of the phenolic hydroxyl group through phenolate formation; meanwhile, the H-bond interaction between the nitro-group in 2-nitroglycal and the thiourea catalyst charged it for the high stereoselectivity. *α*-Selective glycosylation of 2-nitroglucal is difficult by conventional methods; double activation by a bifunctional thiourea **153** could solve this problem, therefore, coupling 2-nitroglucal **162** with **145**, **163,** and **164** provides the desired products **165**–**167** in 75% to 95% yield with *α*:*β* selectivity ranging from 7:1 to 20:1 at −20 °C (Scheme 20).

**Scheme 21:**
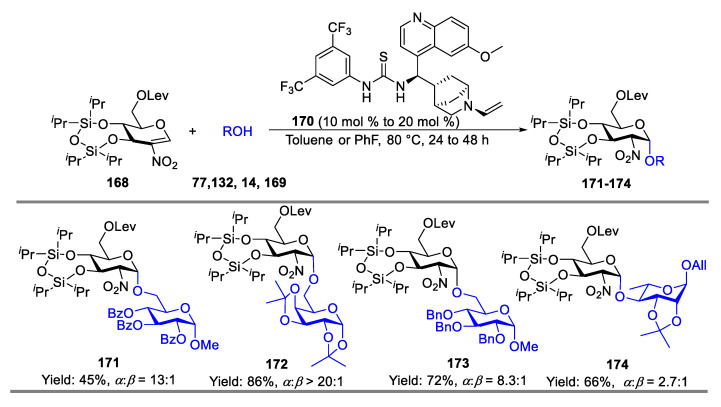
Thiourea **170**-catalyzed glycosylation of 2-nitroglycal **166**.

**Scheme 22:**
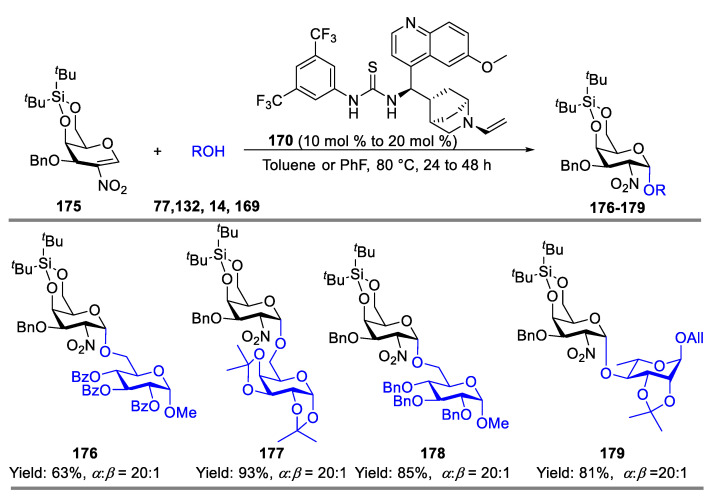
Thiourea **170**-catalyzed glycosylation of 2-nitrogalactal **175**.

Using conformation-constrained 2-nitroglycal as donors and a bifunctional thiourea catalyst, Sun and coworkers recently described a new protocol for the highly efficient and stereoselective production of 1,2-*cis*-2-amino-2-deoxy-glycosidic linkages [64]. Notably, 2-nitroglucal donors, which are prone to provide glycosylation products with lower *α*-stereoselectivity than their 2-nitrogalactal counterparts by using conformation-constrained strategy, delivering the disaccharides in high yield with good-to-excellent stereoselectivity, highlighting the beneficial effect of the cyclic protecting group on stereoselective glycosylation of 2-nitroglucals. For example, glycosylation of conformation-constrained 2-nitroglucal donor **168** with acceptors **77**, **132**, **14**, and **169** in the presence of thiourea catalyst **170** provided the desired disaccharides **171**–**174** in 45–86% yields with *α*:*β* selectivity ranged from 2.7:1 to 20:1 (Scheme 21). Similarly, 2-nitrogalactal **175** as donor also provided *α*-glycoside as the major products, the cyclic di-*tert*-butylsilylidene group in **175** steered the glycosylation reaction to proceed with excellent *α*-stereoselectivity, regardless of acceptors with primary or secondary alcohols (Scheme 22).

**Scheme 23:**
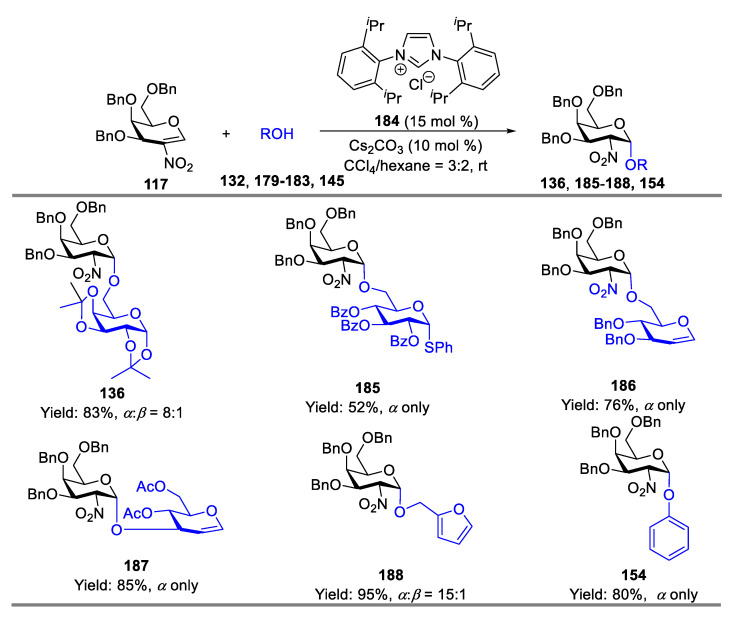
NHC-catalyzed stereoselective glycosylation of 2-nitrogalactals with alcohols and phenol.

In recent years, *N*-heterocyclic carbene (NHC) has emerged as a powerful organocatalyst in organic chemistry [65,66,67,68,69,70]. The key premise in NHC catalysis is the formation and the involvement of an enaminol intermediate, known as the Breslow intermediate [69]. Additionally, NHC-catalyzed reactions through noncovalent bonding interactions have also been developed [71,72,73,74]. Chen and co-workers reported the first NHC catalyzed stereoselective glycosylation of 2-nitrogalactals with alcohols and phenol [62]. A variety of functional groups in the acceptors were compatible in this approach, and the corresponding products were obtained in good-to-excellent yields (52–85%) with high-to-excellent *α*-selectivity (*α*:*β* = 8:1 to *α* only) (Scheme 23).

In 2022, Zhang and co-workers reported stereoselective synthesis of 1,1′-2-amino thiodisaccharides using 2-nitroglycals as donors catalyzed by bifunctional organothiourea [75]. Very recently, the methodology for the regio- and stereo-selective convergent synthesis of 2-amino-2-deoxy-dithioglycosides from 2-nitroglycals was also disclosed by the same group [76].

### 2.3. Glycosylation with Ring-Fused 2,3-Oxazolidinone Thioglycosides

**Scheme 24:**
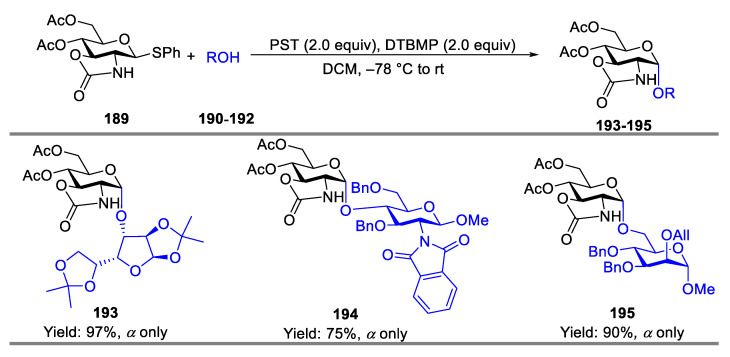
Ring-fused oxazolidinone for the stereoselective formation of *α*-linked glycoside.

In 2001, Kerns and coworkers reported a protocol using 2,3-*trans*-oxazolidinone as an effective protective group with high *α*-selectivity toward glycosylation. A notable feature of this class of *α*-selective D-glucosaminyl donors is that the oxazolidinone group affords simultaneous protection of the 2-*N* and 3-*O* positions [77]. Coupling donor **189** with acceptors **190**–**192** in the presence of PST (phenylsulfenyltriflate, PhSOTf) and 2,6-Di-*tert*-butyl-4-methylpyridine (DTBMP) gives the *α*-linked glycoside **193**–**195** in 75–97% (Scheme 24).

**Scheme 25:**
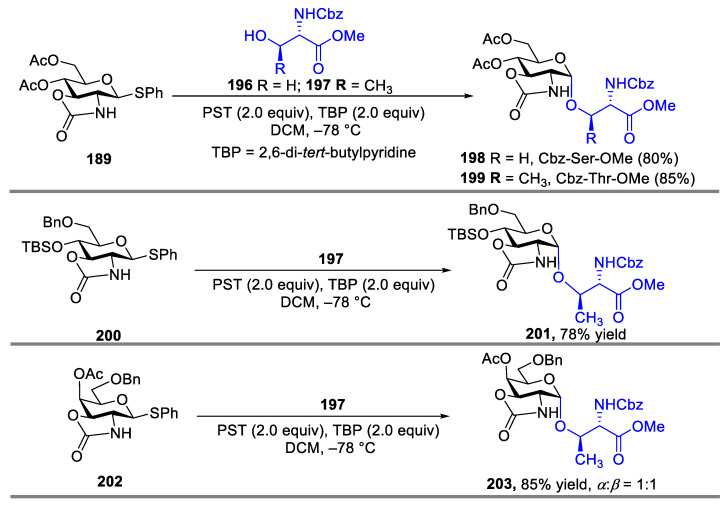
The formation of *α*-linked serine and threonine derivatives.

Later, the same group expanded this methodology to the formation of *α*-linked serine and threonine derivatives [78]. No matter what the protective groups on the sugar were, the desired products could be obtained in high yield (78–85%) with exclusive *α*-selectivity (Scheme 25), however, when the similar reaction was performed with fused-ring 2,3-oxazolidinone galactosamine donor, poor stereoselectivity was observed. Even the glycosylated production was isolated in high yield. The same poor selectivity was also observed with other 2-azido-2-deoxy-D-galactosyl donors (sulfoxide, thiophenyl, and selenoglycoside donors) in the previous reports [79,80,81].

**Scheme 26:**
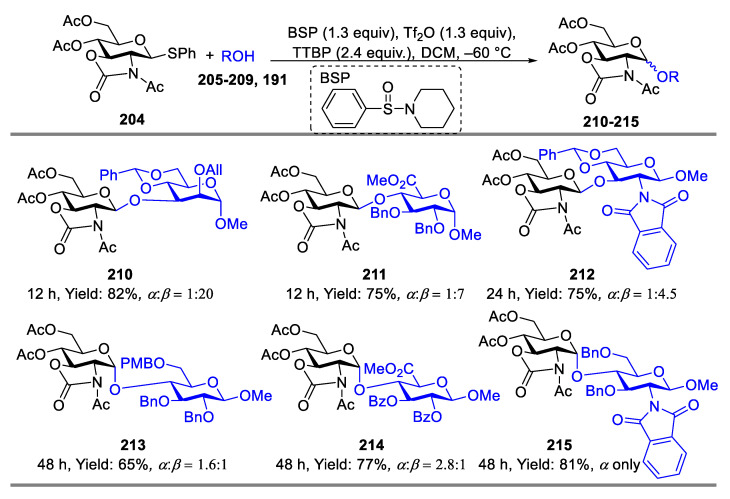
The glycosidation of donor **204** under BSP/Tf_2_O activation conditions.

In 2004, Kerns’s group modified the oxazolidinone group to its *N*-acetyl analogues, leading to switch the reaction from *α*-selectivity to *β*-selectivity [82]. In Kerns’s case, stereoselective formation of *α*-linked or *β*-linked glycoside was supposed to be dependent on the reactivity and steric hindrance of acceptors. Due to the steric hindrance from the acetyl group on *N*-atom, and the equilibrium between *α*-triflate (a major product but with less reactivity) and *β*-triflate (a minor product but with high reactivity), the more-reactive and less-steric-hindered alcohol reacted rapidly with *α*-triflate to produce *β*-linked glycoside; conversely, less-reactive alcohols with steric hindrance could react with minority but more-reactive *β*-triflate to obtain *α*-linked glycoside with extended reaction time (Scheme 26).

**Scheme 27:**
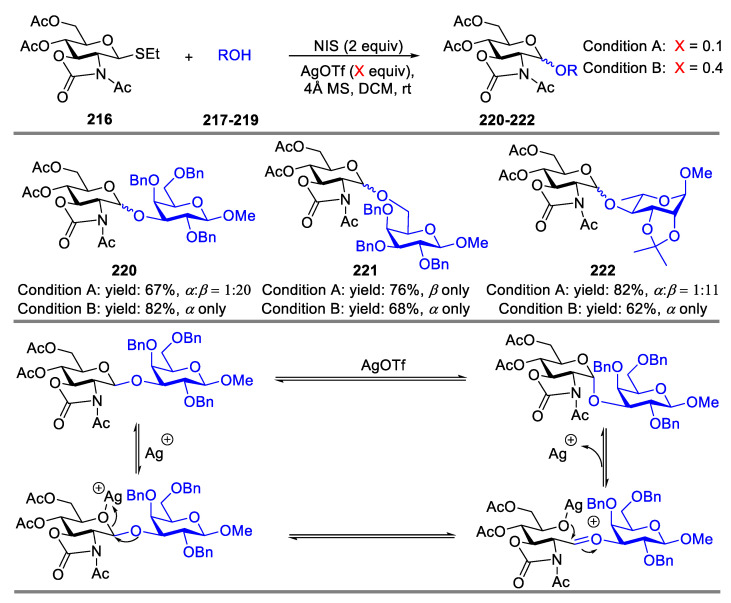
Glycosylation reactions under two different conditions.

In 2005, Stefan Oscarson and co-workers found that when *N*-acetylated 2*N*,3*O*-oxazolidinone thioglycoside was activated with NIS and AgOTf in the presence of glycosyl acceptors, the corresponding glycosylation products could be formed in excellent yield [83]. Interestingly, the stereochemistry of the anomeric carbon could be switched by controlling the amount AgOTf. When the catalytic amount of AgOTf was used, the reaction delivered the products in exclusive *β*-selectivity (Scheme 27, condition A), while 0.4 equiv. of AgOTf resulted in forming the products in total *α*-selectivity (Scheme 25, condition B). A mechanism study has shown that *β*-coupling products could take place in situ anomerization to form *α*-glycosylation products through Ag cation-promoted endocyclic C-O bond cleavage intramolecular cyclization [84].

**Scheme 28:**
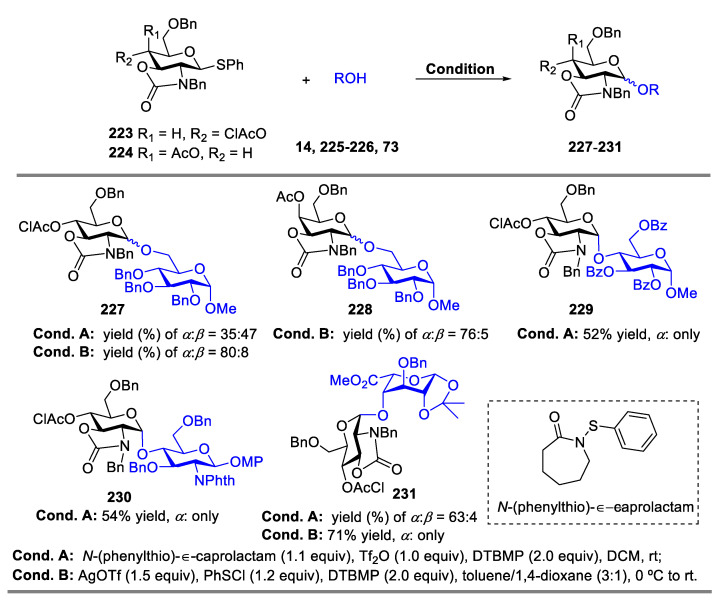
Stereoselectivities of glycosylation reactions using the novel glycosyl donor.

**Scheme 29:**
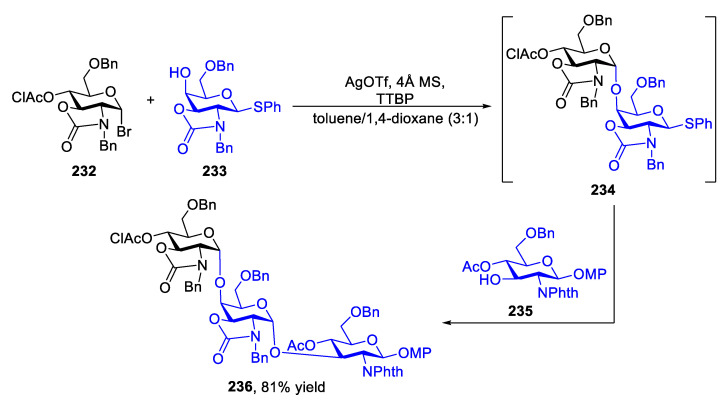
The preparation of trisaccharide **236**.

In 2008, Ito and co-workers reported a stereoselective *α*-glycosylation procedure using *N*-benzyl-2*N*,3*O*-oxazolidinone thioglycoside as a highly *α*-directing glycosyl donor [85]. It was found that the glycosylation reaction between thioglycoside donors **223**–**224** and primary alcohols produced the products in lower selectivity, however, the stereoselectivity could be improved by using toluene and 1,4-dioxane as mixture solvent, meanwhile, a similar reaction with less-reactive secondary alcohol could produce the products in high selectivity regardless of solvents (Scheme 28). The key features of this reaction included: (i) the reaction was performed at room temperature and could be run on large scale; and (ii) the procedure could be used for the preparation of trisaccharide with two 1,2-*cis*-glycosidic bonds by one-pot two-step sequential glycosylation reaction (Scheme 29), which rendered it possible to use in polymer-supported or automated oligosaccharide synthesis.

**Scheme 30:**
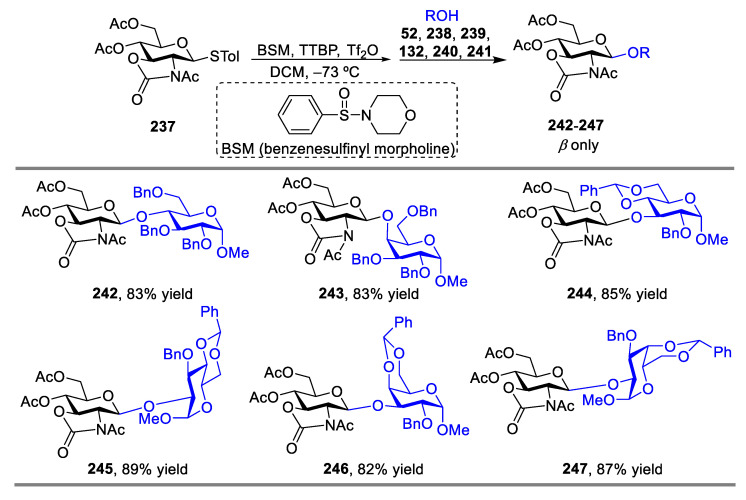
The glycosylation of **237** with acceptors by pre-activation in the presence of TTBP.

**Scheme 31:**
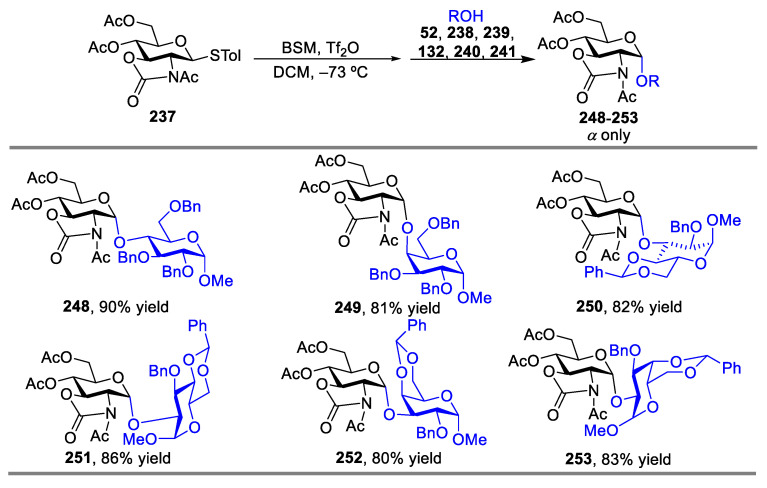
The glycosylation of **237** with acceptors by pre-activation in the absence of TTBP.

Although great advances in this field have been achieved by use of 2,3-*trans*-oxazolidinone as a non-participating group for glucosamine donors, several drawbacks, such as the undesired glycosylation and sulfenylation of the nitrogen atom [77,78], reduction of *α*-selectivity [82], low yields [85], and limited scope of acceptors [84,85], still existed. To tackle these problems, a new efficient strategy based on pre-activation protocol for both *α*- and *β*-stereoselective glycosylation of glucosamine donors was developed by Ye’s group [86]. In this case, a glycosyl donor such as 4,6-di-*O*-acetyl-*N*-acetyl-oxazolidinone protected donor **237**, was completely activated and consumed first, then a glycosyl acceptor was added. By using this strategy, either excellent *α*-stereoselective or *β*-stereoselective glycosylation could be achieved in the absence of any base or by the addition of a hindered base, such as 2,4,6-tri-*tert*-butylpyrimidine (TTBP) [87] (Scheme 30 and Scheme 31). Compared with the traditional glycosylation protocols, controllable glycosylation could be realized by simply adding a steric-hindered base or without addition of any base under pre-activation procedure, which facilitated the construction of complex oligosaccharides with important biological functions. The same group also discovered that the protected group in the oxazolidinone donors could dramatically influence the stereoselectivities of the pre-activation procedure [88].

### 2.4. Glycosylation with C(2)-Benzylidenamino Donors

#### 2.4.1. The Development of Nickel-Catalyzed Stereoselective Glycosylation

**Scheme 32:**
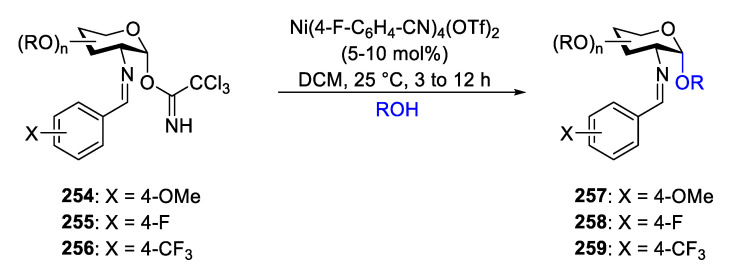
Nickel-catalyzed stereoselective formation of 1,2-*cis*-aminoglycoside.

The Nguyen’s group focused on the development of novel, scalable approaches to avoid the issues associated with the formation of 1,2-*cis*-2-aminoglycoside by using cationic nickel (II) catalysis [89,90]. Trihaloacetimidate (TCA) is viewed as a good “leaving group” in carbohydrate chemistry whose nitrogen can be coordinated with metals to play a role as a “directing group” in transition-metal-catalyzed reactions. Additionally, the benzylidene at C(2)-amino group of sugars serves not only as a “protecting group” but also as a “directing group” in transition-metal-catalyzed reactions [91]. In 2009, The Nguyen’s group reported a nickel-catalyzed stereoselective glycosylation with C(2)-*N*-para-(methoxy)benzylideneamino donor **254** for the formation of 1,2-*cis*-2-aminoglycoside (Scheme 32) [89]. The thorough adjustment of the reaction conditions revealed that the catalyst, electronic properties of the ligands, and the substituted group on the benzylidenamino protecting-group all played crucial roles in regulating the stereoselectivity and speeding up the reaction.

**Scheme 33:**
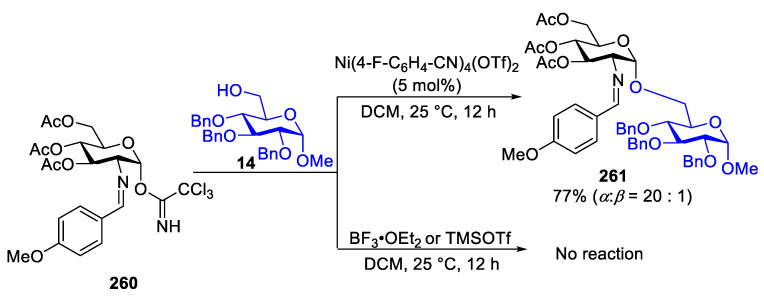
Comparison between Lewis acids and nickel catalyst.

In order to prove the superiority of the catalytic system in the glycosylation reaction, comparisons against conventional trichloroacetimidate donor-activation protocol and the developed nickel-catalyzed formation of 1,2-*cis*-aminosugar was performed. Classical Lewis acid activator of TCA donor, such as TMSOTf and BF_3_·OEt_2_, either resulted in the decomposition of the glycosyl donor or led to the formation of a trace amount of the desired disaccharide **261** [92] (Scheme 33). Compared to these established glycosylation methods [88,92], the coupling of **260** with **14** proceeded smoothly to provide disaccharide **261** in 77% yield with excellent *α*-selectivity.

**Scheme 34:**
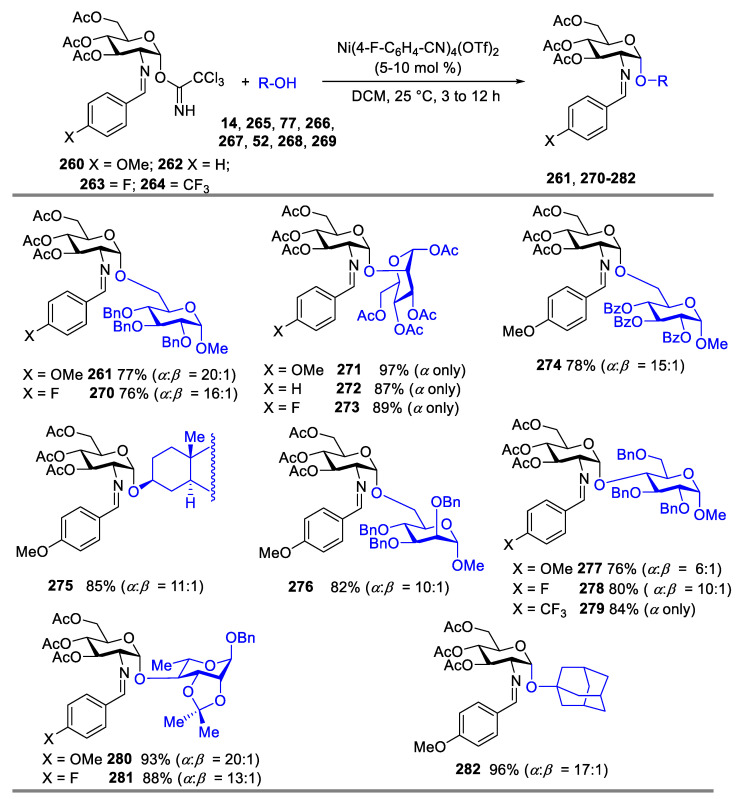
Substrate scope of nickel-catalyzed 1,2-*cis*-amino glycosylation.

Glucosamine donors **260**, **262**–**264** were then reacted with a broad range of glycosyl acceptors, such as primary, secondary, and tertiary alcohols (Scheme 34). All the glycosylations with cationic nickel(II) catalyst produced the desired products **261**, **270**–**282** in high yield with excellent *α*-selectivity. The donor with steric hinderance, such as tertiary adamantol, also produced the desired product **282** in 96% yield and with *α*-selectivity (*α*:*β* = 17:1). Dihydrocholesterol **266**, a secondary alcohol acceptor, was employed to couple with the donor **260** to produce glycoconjugate **275** in 85% yield with *α*-selectivity (*α*:*β* = 11:1). Increased yield and *α*-selectivity are observed when glucosamine donors are combined with acceptor **260** when the *para*-substituent on the benzylidene group is simply changed from *p*-OMe-benzylidene in **277** (*α*:*β* = 6:1) into *p*-CF_3_-benzylidene (*α* only). Meanwhile, it was found that electron-withdrawing C(2)-*N*-*para*-fluoro- and trifluoromethyl-benzylidenamino donors **263** and **264** are more stable compared to the methoxy donor **260** for purification [62], which would facilitate it for preparation on a large scale.

**Scheme 35:**
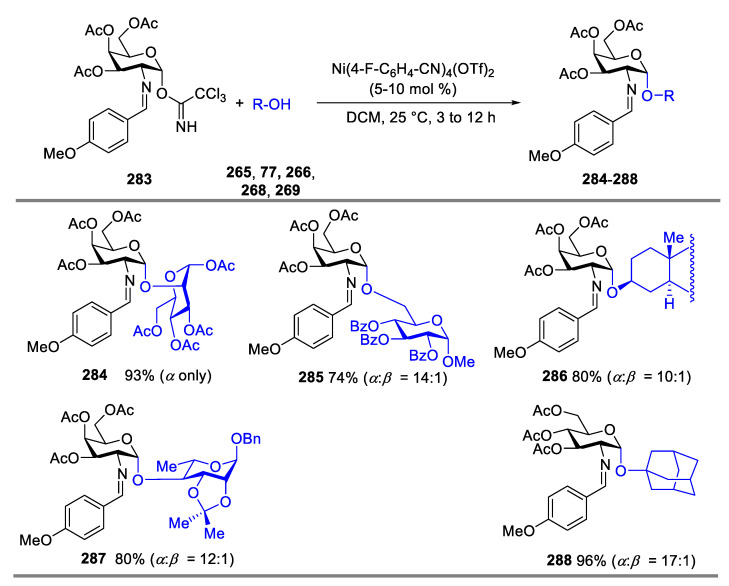
*α*-Selective coupling with D-galactosamine trichloroacetimidate.

Additionally, galactosamine trichloroacetimidate **283** was also employed as a donor to couple with both primary and secondary alcohol acceptors (Scheme 35). Once again, these results showed the cationic nickel (II) catalyst in the glycosylation reaction could produce 1,2-*cis*-2-aminoglycosides in high yield with outstanding *α*-selectivity regardless of the kind of the acceptors.

**Scheme 36:**
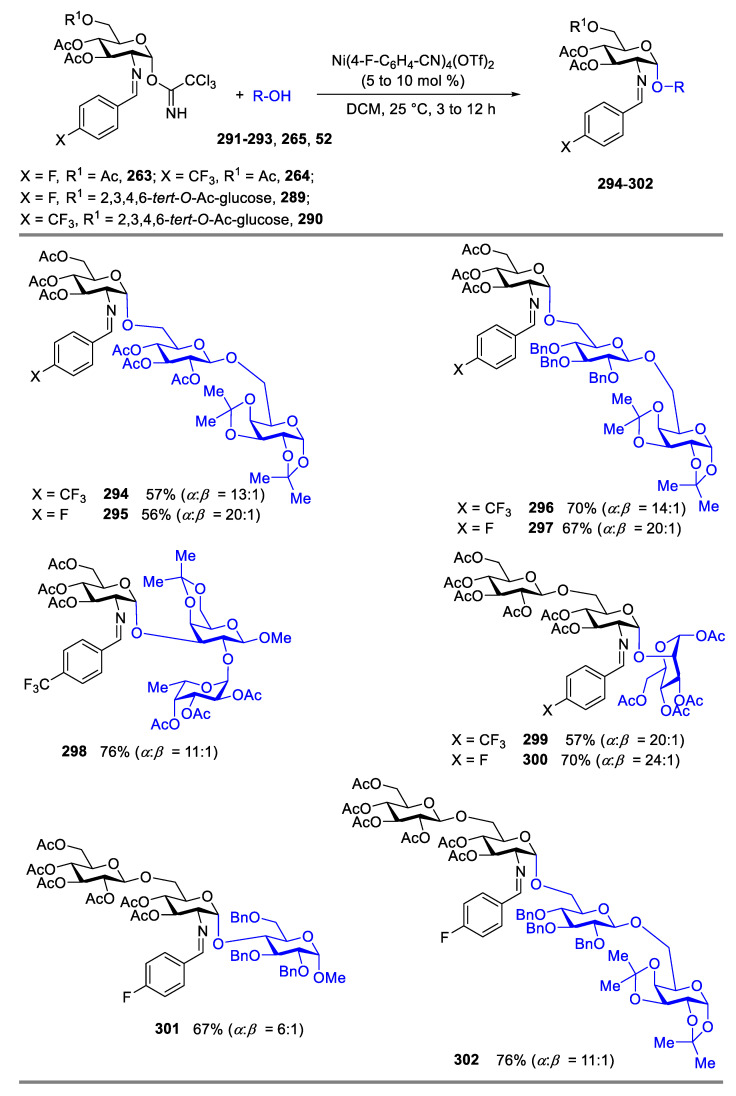
*α*-Selective glycosylation of disaccharide acceptors.

Additionally, the nickel method was also suitable for oligosaccharide synthesis by (1 + 2), (2 + 1), or (2 + 2) process (Scheme 36). In a (1 + 2) strategy, the electron-rich disaccharide acceptors gave a higher yield than the electron-deficient one, however, the selectivity of these two acceptors is almost uniform (**294** vs. **296**, and **295** vs. **296**). Coupling the secondary alcohol of disaccharide acceptor **292** with donor **264** provides the desired trisaccharide **298** in 76% yield with a high *α*-selectivity (*α*:*β* = 11:1). In a (2 + 2) strategy, coupling disaccharide acceptor **292** with disaccharide donor **289** proceeded smoothly to provide tetrasaccharide **302** in 76% yield with *α*-selectivity (*α*:*β* = 11:1).

**Scheme 37:**
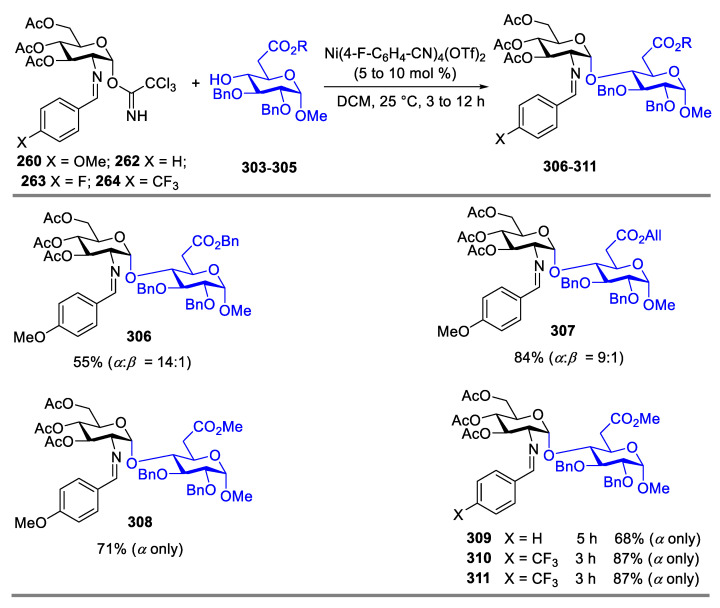
*α*-Selective glycosylation of glucuronic acid acceptors.

Due to the lower reactivity, using D-glucuronic acid derivatives as acceptors to couple with donors in a glycosylation reaction generally gave the products in low yield and poor selectivity. However, the nickel-catalyzed strategy might be able to partially resolve this issue [93,94] (Scheme 37). For instance, coupling acceptors **303**–**305** with donors **260**, **262**–**264** proceeded smoothly to provide the desired disaccharides in moderate-to-good yields (55–87%) with good *α*-selectivity (*α*:*β* = 9:1 to *α* only). Among different benzylidenamino protected donors (**260**, **262**–**264**), the one with an electron-withdrawing group, such as *para*-(trifluoromethyl)benzyl (**264**), was found to be the most efficient. Glycosylation of the methyl ester of D-glucuronic acid acceptor **305** with donor **264** produced the desired disaccharide **311** in 87% yield with exclusive *α*-selectivity.

**Scheme 38:**
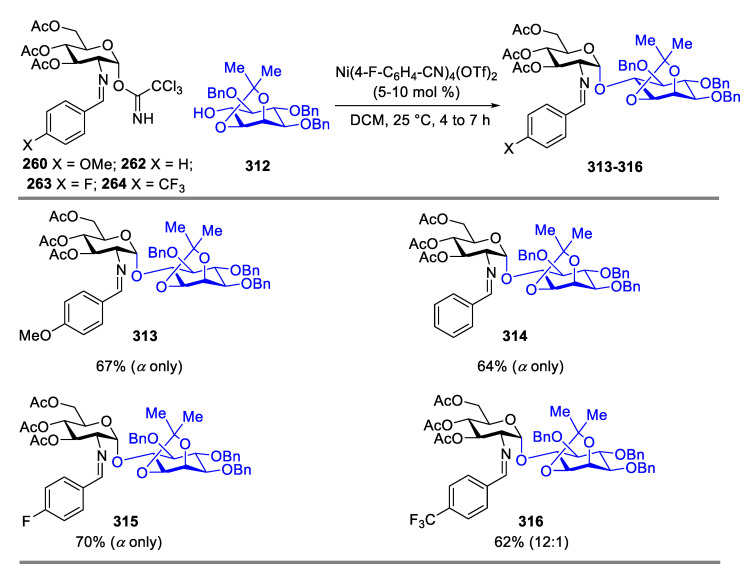
*α*-Selective glycosylation of inositol acceptor.

Glycosylphosphatidylinositol (GPI) anchors are a large family of glycolipids that play significant roles in various biological processes, including signal transduction, prion disease pathogenesis, and immune response [95,96,97,98,99]. Total synthesis of GPI anchors is challenging due to structural complexity. The available methods of construction of the pseudodisaccharide unit of D-glucosamine and inositol components had several limitations, such as poor *α*-selectivity and long reaction time [100,101,102,103,104]. Coupling inositol nucleophile **312** as the acceptor with C(2)-*N*-substituted benzylidene D-glucosamine TCA donors **260**, **262**–**264** produced desired products **313**–**315** in excellent *α*-selectivity (Scheme 38), addressing the issue of poor *α*-selectivity in the literature.

**Figure 4:**
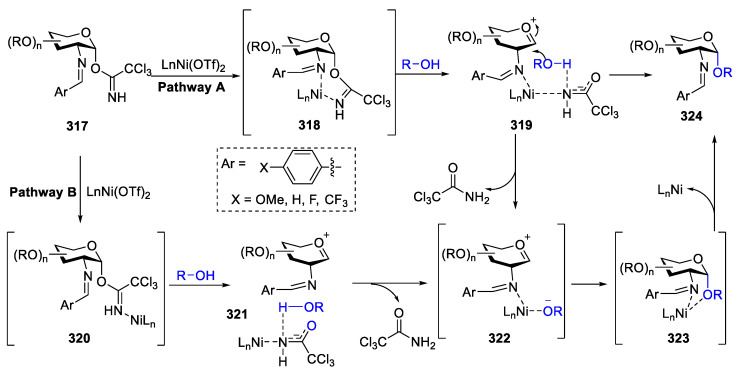
The proposed mechanism for nickel-catalyzed 1,2-*cis*-2-amino glycosylation.

A mechanism for explaining *α*-selectivity of the glycosylation with cationic nickel (II) catalyst was shown in Figure 4. In pathway A, the formation of seven-member ring intermediate **318** via reversible coordination of C(2)-benzylidene nitrogen and the nitrogen of the C(1)-TCA donor **317** by nickel catalyst facilitated the 1,2-*cis*-glycoside. Alternatively, the cationic nickel catalyst could act as a mild Lewis acid (pathway B) to activate C(1)-TCA donor **317,** promoting ionization to generate the oxocarbenium intermediate **321**. Subsequently, the formation of five-membered cyclic intermediate **323** was followed by ligand exchange **322,** providing the 1,2-*cis*-glycoside **325**.

**Scheme 39:**
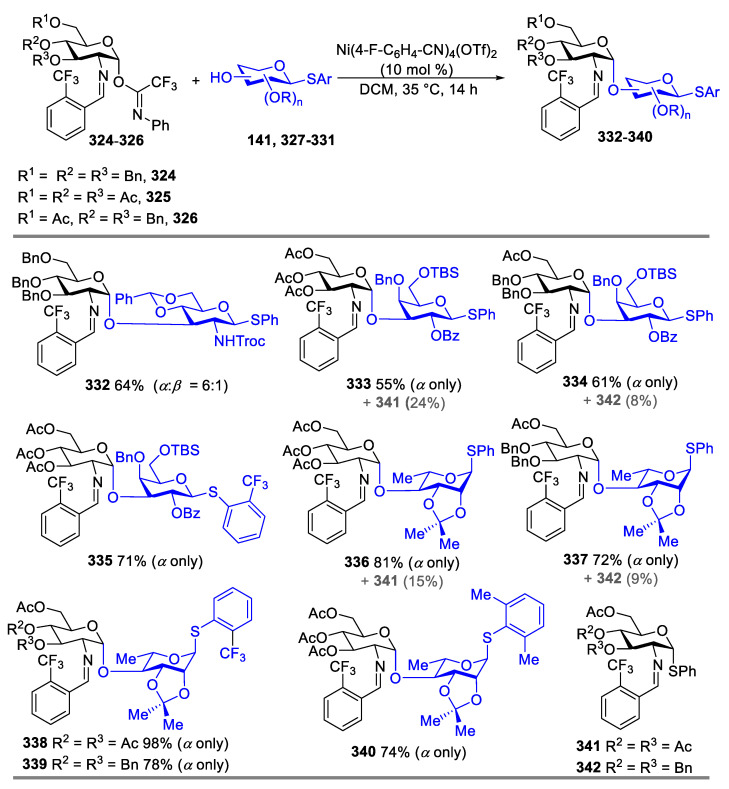
Coupling of various thioglycosides with *N*-phenyl trifluoroacetimidate donors.

The potential of the nickel-catalyzed glycosylation strategy was further demonstrated by extending it to couple with thioglycoside acceptors. A key feature for synthesis of thioglycoside was that it was easily synthesized and generally stable under a variety of glycosylation conditions, making it compatible with other glycosylation strategies, which, in turn, facilitated it for the multistep synthesis of oligosaccharides and glycoconjugates. However, using thioglycoside as glycosyl acceptor under nickel catalyst is much more of a challenge because: (i) intermolecular aglycon transfer of the anomeric sulfide to the trichloroacetimidate donor could result in undesired thioglycoside [105,106,107,108,109,110,111,112,113,114,115,116,117,118,119,120]; and (ii) sulfur could coordinate to the cationic nickel catalyst leading to its inactivation. However, after a series of trials, the desired disaccharide products were obtained in good yields with excellent *α*-selectivity, and only a minor mount of undesired thioglycoside was detected. The key for the success of this transformation was using *N*-phenyl trifluoroacetimidate (PTFA) as donors due to their attenuated activity compared to trichloroacetimidates [121,122,123]. The scope of substrates was then expanded to the glycosylation of a variety of thioglycosides with both armed and disarmed donors [124] (Scheme 39). Three L-rhamnose acceptors equipped with different anomeric sulfides (**329**–**331**) were coupled with corresponding donors (**324** and **325**). Coupling of armed donor **324** with phenyl thioglycoside **141** generates disaccharide **332** in 70% yield with no undesired thioglycoside by-product was detected, albeit in lower *α*-selectivity (*α*:*β* = 6:1). Similarly, coupling of phenyl D-galactosyl thioglycoside **327** with both donors **325** and **326** (entries 2 and 3) provids disaccharides **333** and **334** in exclusive 55% and 61% yield with exclusive *α*-selectivity but a large quantity of sulfide transfer products **341** and **342** were detected (8 vs. 24%), respectively. The donor **325** then reacted with D-galactosyl thioglycoside **328**. No trace amount of transfer product **341** was observed, and exclusive *α*-disaccharide **335** was isolated in 71% yield. The desired disaccharides **336** and **337** were obtained in 81% and 72% yield with exclusive *α*-selectivity, also aglycon transfer products **341** and **342** were detected in 15% and 9% yield, respectively. Compared to the acceptor **330**, the coupling of bulky 2,6-dimethylphenyl (DMP) [125] acceptor **331** with donor **325** produced the desired product **340** in a slight lower yield (98% vs. 74%) with the *α*-only selectivity maintained but no undesired thioglycoside by-product was detected. Additionally, 2-trifluoromethylphenyl thioglycoside acceptor **330** and galactosamine acceptors **327** and **328** were also investigated, providing *α*-disaccharides, and aglycon transfer products were not observed. Galactosamine donors were also employed to couple with corresponding acceptors, but the desired products were obtained in lower yield and minor amounts of undesired thioglycosides were observed in some reactions.

**Scheme 40:**
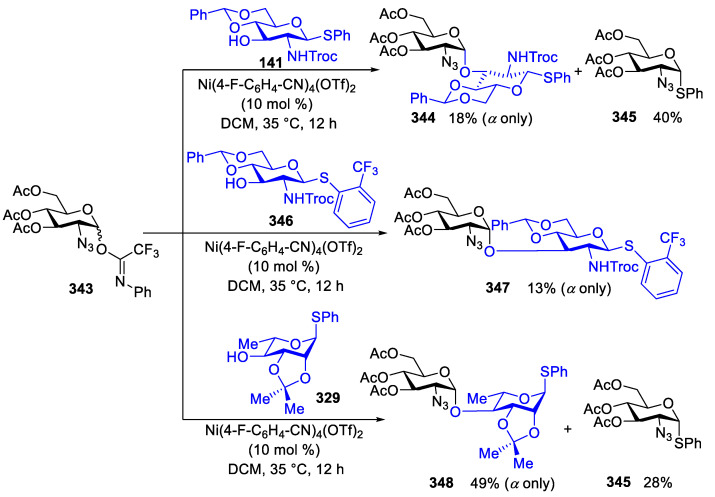
Glycosylation with C(2)-azido imidate donor.

To compare, the author also performed nickel-catalyzed glycosylation using C(2)-azido of *N*-phenyltrifluoroacetimidate as the glycosyl donor. It was found that the yield of disaccharide products decreased dramatically but *α*-only selectivity was maintained (Scheme 40). In the same case, such as with **141** as glycosyl acceptor, the major product of the reaction was thioglycoside by-product derived from the intermolecular aglycon transfer of the anomeric sulfide to *N*-phenyltrifluoroacetimidate.

**Scheme 41:**
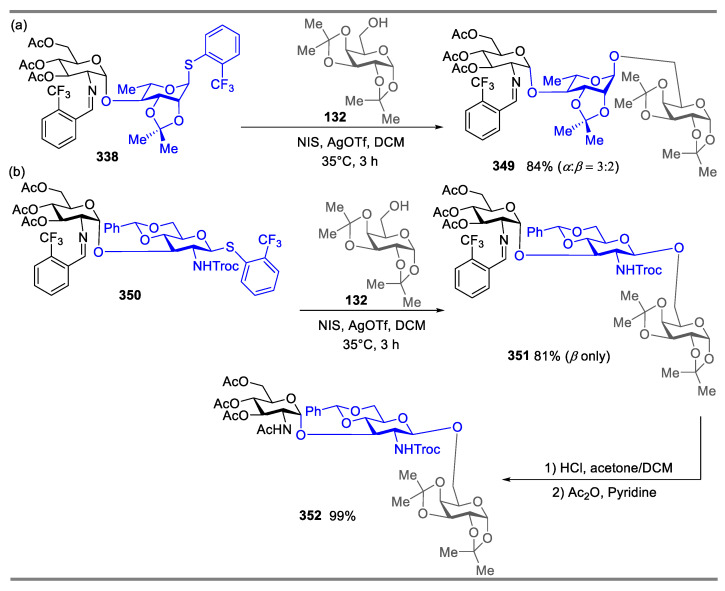
(**a**) The synthesis of trisaccharide **349**; (**b**) The synthesis of trisaccharide **352**.

To further explore the utility of this method, the sequential formation of trisaccharides was performed (Scheme 41). Coupling disaccharide thioglycoside **338** obtained from nickel-catalyzed glycosylation with acceptor **132** under NIS-AgOTf activation condition at 35 °C produced trisaccharide adduct **349** in 84% yield with the a slight *α*-selectivity (*α*:*β* = 3:2). Similarly, trisaccharide **351** was obtained by coupling disaccharide thioglycoside **350** with acceptor **132** under the same conditions. Subsequent deprotection of imine by treatment of **351** with HCl, acetone/DCM, followed by acetylation in the presence of acetic anhydride and pyridine provided corresponding product **352** in quantitative yield.

#### 2.4.2. Synthesis of Biologically Active Glycans via Nickel-Catalyzed Glycosylation

Nguyen’s group further extended nickel-catalyzed glycosylation to synthesis of several biologically relevant glycans and glycoconjugates including mycothiol, precursors of GPI anchor and T_N_ antigen.

##### Synthesis of Mycothiol

**Figure 5:**
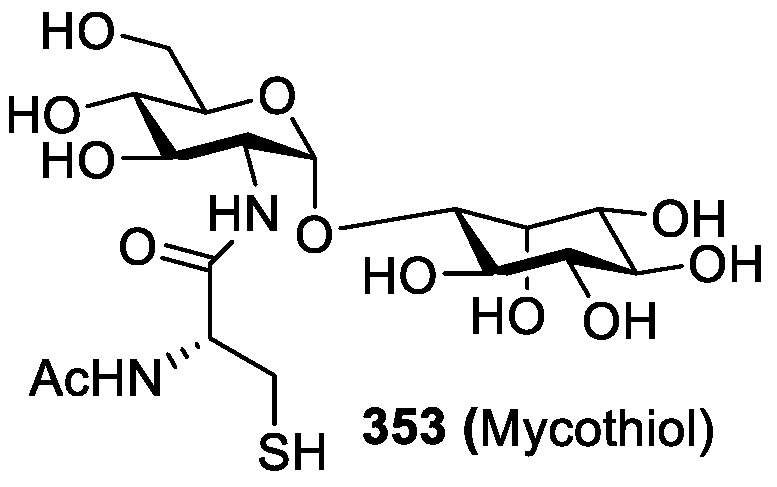
The structure of mycothiol.

Mycothiol (MSH, **353**) (Figure 5) was first isolated as a dimer (polymerized by disulfide bond) by Yasuhiro Yamada from *Streptomyces* sp. AJ9463 in 1994. It is a low-molecular-weight thiol composed by an unusual *N*-acetylcysteine amide-linked to 1-D-myo-inosityl-2-amino-2-deoxy-*α*-D-glucopyranoside (GlcN-*α*(1-1)-Ins). MSH is used for protection of actinomycetes against foreign electrophilic reagents, such as oxidants and radicals [126,127,128]. Most actinomycetes produced MSH as the major thiol, but at millimolar levels, and this limited its application in the study on the function of MSH, which, in turn, promoted the development of total synthesis of MSH. Previously, except for the intramolecular *α*-glucosaminidation [129] and desymmetrization of meso-inositol strategies [130], the existing chemical synthesis provided the desired pseudo-disaccharide of MSH in poor-to-moderate selectivity (*α*:*β* = 1:1 to 6:1) [129,130,131,132,133]. Accordingly, the nickel-catalyzed method was employed in the synthesis of mycothiol core pseudosaccharides via coupling a mixture of *α*- and *β*-isomers of C(2)-*N*-substituted benzylidenamino donors with C(1)-hydroxyl inositol acceptor **359** [134].

**Scheme 42:**
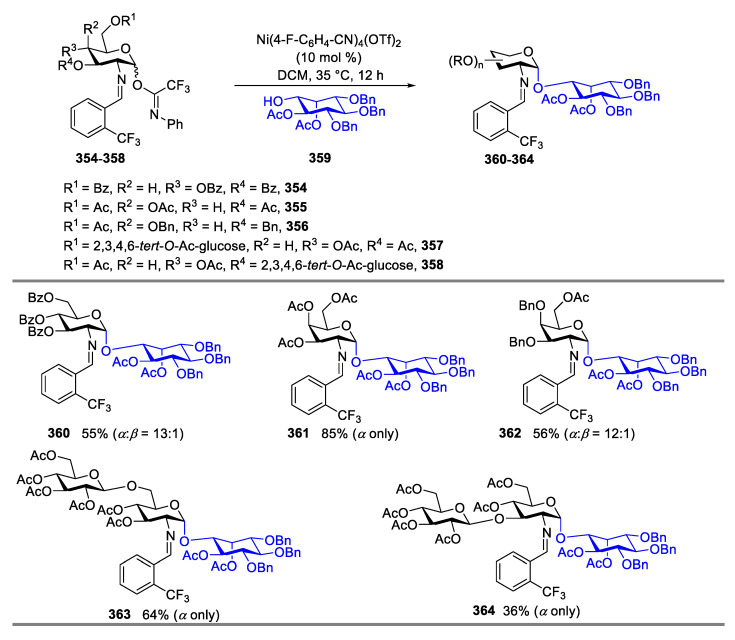
The synthesis of core pseudosaccharides of mycothiol.

As shown in Scheme 42, under standard nickel-catalyzed glycosylation conditions, the mycothiol core analogs were isolated in moderate-to-good yield (36–64%) with excellent selectivity (*α*:*β* = 12:1 to *α* only). When the tribenzoylated substrate **354** was employed as the donor, the anomeric selectivity of the desired product **360** is *α*:*β* = 13:1. Additionally, coupling the galactosamine dibenzylated donor **356** with acceptor **359** generated the desired product **362** in 56% yield with *α*:*β* = 12:1. Even when 1,6-linked disaccharide **357** was employed as donor, the desired pseudotrisaccharide **362** was still produced in 64% yield with exclusively *α*-selectivity. Similarly, coupling 1,4-linked disaccharide **357** with **359** produced pseudotrisaccharide **364** in 64% yield and exclusive *α*-selectivity as well.

**Scheme 43:**
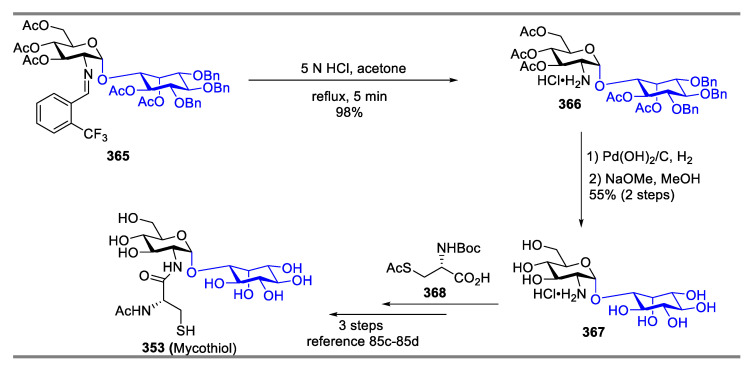
The formation of mycothiol.

To synthesise mycothiol **353**, the 2-trifluoromethyl-benzylidene in pseudodisaccharide **365** was removed in the presence of 5N HCl to obtain free ammonium salt **366**. Hydrogenolysis of **366** with Pd(OH)_2_/C and H_2_ [135], which was followed by deacetylation, generated **367** in 55% yield over two steps. Coupling **367** with cysteine residue **368** formed mycothiol **353** following the previously reported procedures [129,130] (Scheme 43).

##### Synthesis of GPI Anchor

**Scheme 44:**
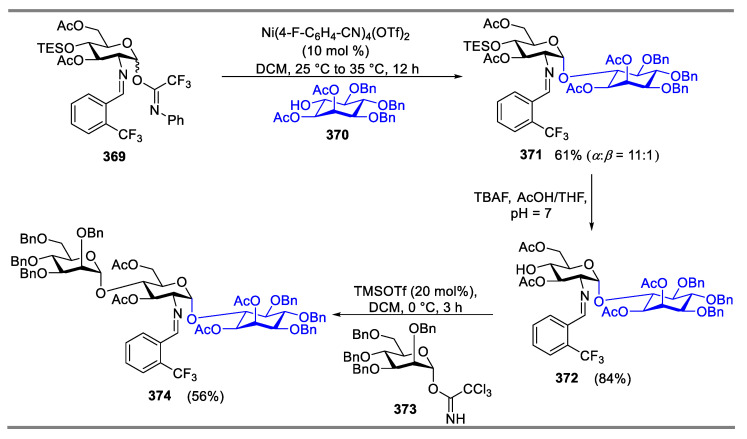
The formation of the GPI anchor core.

The GPI anchor, a glycophospholipid, is post-translationally tethered to a protein for attachment to a cell membrane [136]. These anchored proteins play a pivotal role in biochemical processes, such as signal transduction, immune response, and cancer metastasis [137,138]. All GPI structures share the same basic core, including a phosphoethanolamine linker, a tetrasaccharide attached to the C(6)-position of myo-inositol, and phospholipid tail [139]. Despite the syntheses of GPI anchors have been reported extensively, there are several challenges to stereoselectively form the 1,2-*cis*-glycosidic bond between the glucosamine unit and the myo-inositol component [140,141,142]. The cationic nickel (II) catalysis developed by Nguyen’s group is thought to be suitable for the GPI anchor core due to its similarity with mycothiol **353** (Scheme 44) [139]. Coupling C(4)-TES-C(2)-*N*-benzylidene donor **369** with C(6)-hydroxyl myo-inositol **370** under the standard condition generated the desired pseudodisaccharide **371** in 61% yield with *α*:*β* = 11:1. Subsequent removal of the TES group with TBAF generated the disaccharide **372**. Finally, glycosylation of **372** with tetrabenzylated D-mannose trichloroacetimidate **373** afforded the desired GPI core pseudotrisaccharide **374** in 56% yield.

##### Synthesis of T_N_ Antigen

**Figure 6:**
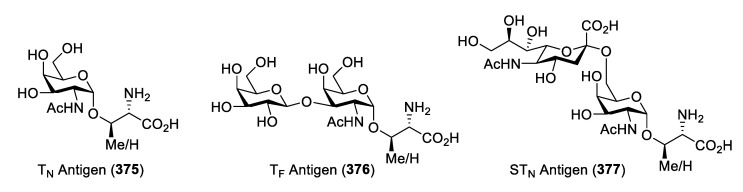
The structure of T_N_, T_F_ and ST_N_ antigens.

T_N_ antigen is a core structure in *O*-linked glycoproteins, which consists of the linkage of an *N*-acetyl-galactosamine (GalNAc) and the hydroxyl group of serine or threonine via *α*-glycosidic bond [143]. T_N_ antigen **375** and some other analogs, such as T_F_ antigen **376** and ST_N_ antigen **377** (Figure 6), are widely distributed on cell-surface mucin glycoproteins, which participate in cell-adhesion events associated with cancer metastasis, having potential as a biomarker or as a vaccine against cancer [144,145,146].

**Scheme 45:**
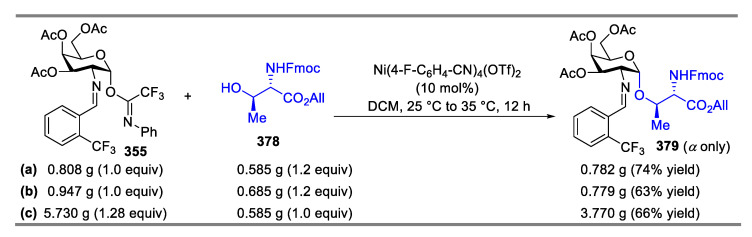
Synthesis of TN antigen core via nickel-catalyzed glycosylation.

Early work employed the C(2)-oxazolidinone and the C(2)-azido as donors to couple with the acceptor, affording a mixture of anomers with the ratio of *α:β* ranged from 1:1 to 4:1 [78,147]. Large-scale synthesis of T_N_ antigen core in good yield with *α*-selectivity could be achieved under the condition of cationic nickel (II) catalysis (Scheme 45). Two small-scale (Scheme 45a,b) and scale-up reactions (Scheme 45c) employing donor **355** were performed by using 10 mol% of Ni(4-F-C_6_H_4_-CN)_4_(OTf)_2_ as the catalyst [148]. These scale-up reactions without loss in yield and selectivity showed that the cationic nickel (II) catalyst might be suitable for formation of 1,2-*cis*-aminoglycoside in large-scale industry production.

**Scheme 46:**
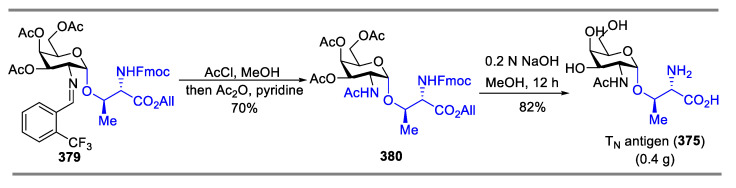
Gram-scale synthesis of T_N_ antigen.

Finally, gram-scale synthesis of T_N_ antigen from **379** was performed (Scheme 46). Treatment of **379** with HCl(g) (in situ formed from the reaction between AcCl and MeOH) followed by the addition of Ac_2_O-generated *N*-acetylated **380**. Full deprotection of **378** with 0.2N NaOH in methanol produced 0.4 g T_N_ antigen **375** in 82% yield. Additionally, the synthesis of T_N_/T_F_ antigens was also completed by Nguyen’s group via the nickel methodology in 2016 [149] and 2019 [150].

## 3. Glycosylation of 3-Amino-3-Deoxysugars and 4-Amino-4-Deoxysugars

**Scheme 47:**
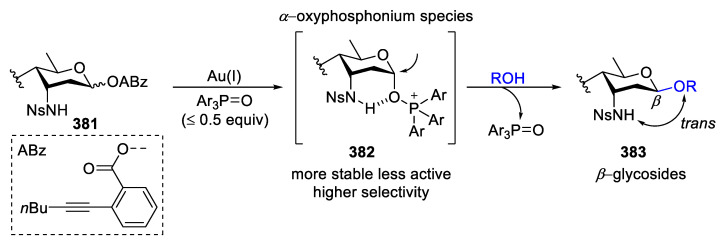
H-bond-stabilized oxyphosphonium species for *β*-glycosylation.

Over the past few years, Wan’s group has been working on the development of novel and effective methodologies for stereoselective formation of 3-aminoglycoside and applying the methods developed by them for the synthesis of natural products containing 3-amino-3-deoxysugars [151,152]. Inspired by their earlier report where an intramolecular hydrogen bond (HB) between a C3-nosyl group and an *α*-hydroxyl group on the anomeric carbon was observed in X-ray crystallography [153], they reported an HB-assisted protocol for *β*-selective glycosylation of rare 3-amino sugars (Scheme 47) [154]. By employment of **381** as the glycosyl donor and phosphine oxide as an exogenous nucleophile, an *α*-oxyphosphonium species **382** was generated, in which there was an intramolecular hydrogen bond between the C3-axial sulfonamide group and the oxygen of the *α*-aligned phosphine oxide. Oxyphosphonium species **382** blocked *α*-face leading to nucleophiles attacked to **382** from *β*-face, which, in turn, resulted in *β*-selective glycosylation.

**Scheme 48:**
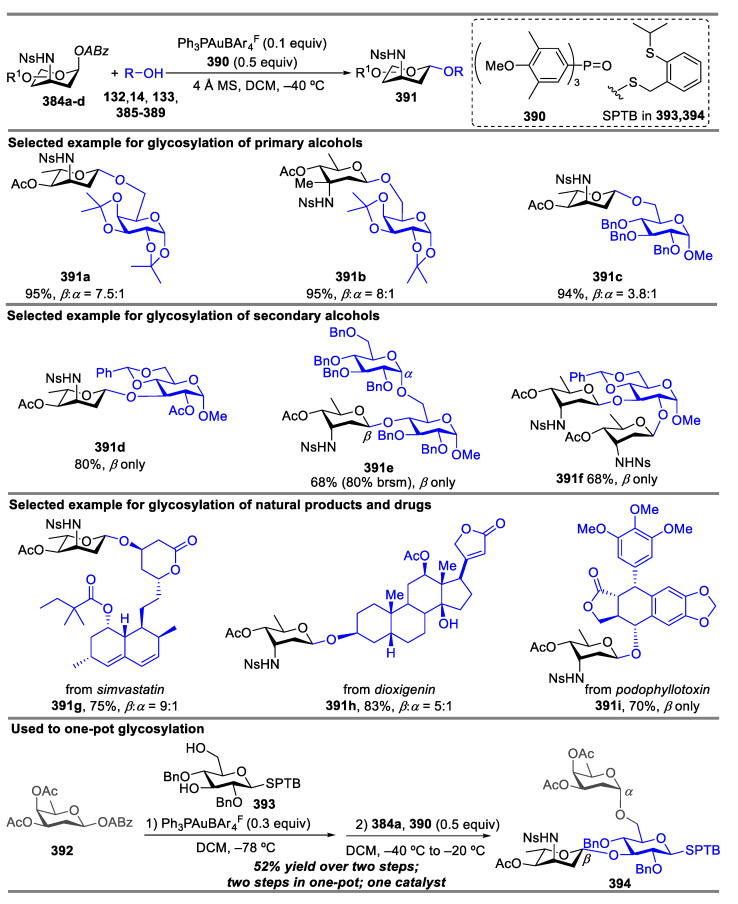
Substrate scope.

This H-bond-assisted glycosylation reaction was found to have broad application in the construction of *β*-glycosidic bonds (Scheme 48). For example, coupling of donor **363a**–**d** with bulky primary alcohol offered glycosylation products in better selectivity than that with the less sterically-hindered and electron-rich primary alcohols (**366a** and **366c**) due to the latter one being a good H-bond acceptor to compete with the phosphine oxide additive. Secondary alcohols generally gave the products with *β*-selectivity. Additionally, this strategy was also extended to modification of natural products and drugs in good yield with satisfactory-to-excellent *β*-selectivity (**391g**–**391i**). This strategy was also suitable for the one-pot formation of trisaccharide such as **394** with Ph_3_PAuBAr_4_^F^ as the only catalyst. The glycosylation reaction between donor **392** and acceptor **393** occurred selectively on the 6-OH of **392** with *α*-selectivity in the presence of Ph_3_PAuBAr_4_^F^ catalyst. Then, **384a** and **390** were added to the resulting mixture, furnishing the **394** with exclusive *β*-selectivity in 52% yield over two steps; impressively, the whole process used Ph_3_PAuBAr_4_^F^ as the only catalyst for the two glycosylation reactions.

**Scheme 49:**
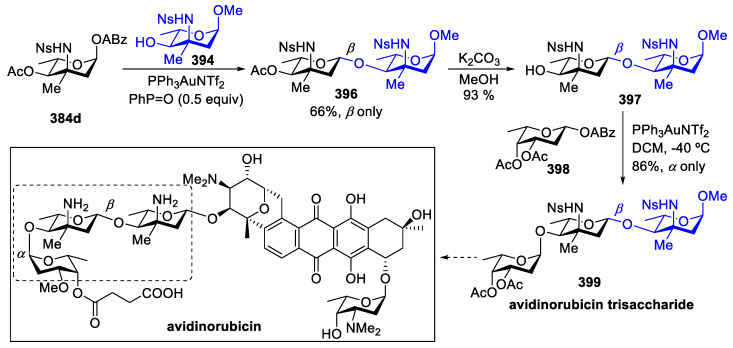
Synthesis of the trisaccharide unit of avidinorubicin.

The methodology was further applied in the synthesis of trisaccharide fragment **399** of avidinorubicin. Avidinorubicin is an antibiotic isolated from the cultured broth of Streptomyces avidinii in 1991, it exhibited platelet-aggregation-inhibitory activity [155]. The synthesis of avidinorubicin is challenging due to its complex structure. Coupling 3-ADS **384d** with **394** produced the desired disaccharide **396** in 66% yield with exclusive *β*-selectivity. Subsequent removal of the acetyl group generated **397** in 93% yield. Finally, glycosylation between 2,6-dideoxy sugar **398** and disaccharide **397** under Yu’s glycosylation conditions proceeded smoothly, generating the protected trisaccharide unit of avidinorubicin **399** in 86% yield with exclusive *α*-selectivity (Scheme 49).

**Scheme 50:**
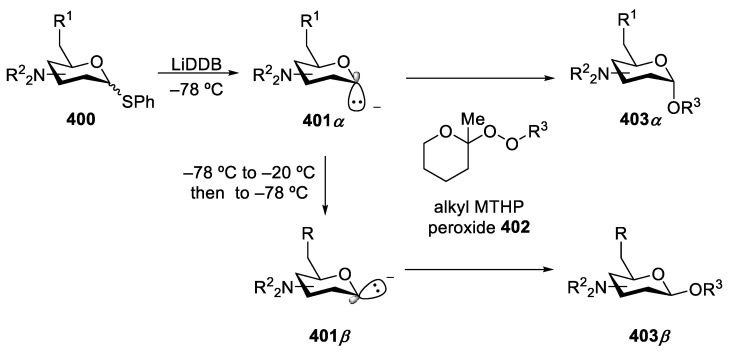
Anomeric anion approach to aminoglycoside.

Herzon’s group reported the synthesis of deoxyaminoglycoside bearing basic amine residues in 2021 [156], based upon their early work on the synthesis of 2-deoxyglycoside by similar strategy in 2019 [157]. In this strategy, they effectively used equilibration between *α*-anomeric anion and *β*-anomeric anion by adjusting the reaction temperature. Lithium 4,4′-di-*tert*-butylbiphenylide (LiDBB) [158] was employed in reductive lithiation of thiophenyl glycoside **400** to form the axial (*α*) anion **401*α*** as the kinetic product [159,160,161,162], then alkyl (2-methyl)-tetrahydropyranyl (MTHP) peroxide **402** [163,164] was added to the reaction mixture, resulting in alkoxenium ion transfer to generate the *α*-linked glycoside **403*α***. Warming the *α* anion to −20 °C followed by re-cooling to −78 °C with peroxide **404** addition formed products **403*β*** in *β*-selectivity, due to the thermodynamically more stable [165] equatorial (*β*) anion **401*β*** rather than the axial (*α*) anion **401*α*** (Scheme 50).

**Scheme 51:**
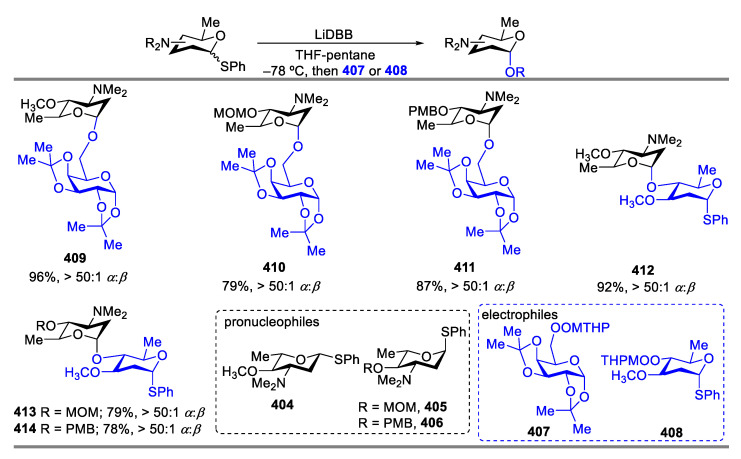
The synthesis of *α*-aminoglycoside.

As for the scope of substrates, the synthesis of *α*-aminoglycoside was investigated first (Scheme 51). Coupling the acosamine derivatives **404**–**406** with the primary MTHP electrophile **407** generates the desired disaccharides **409**–**411** in 79−96% yield with *α*-selectivity (*α*:*β* > 50:1). The secondary MTHP electrophile **406** was also employed to react with these pronucleophiles, producing the desired disaccharides **412**−**414** in 78−92% yield with *α*-selectivity (*α*:*β* > 50:1).

**Scheme 52:**
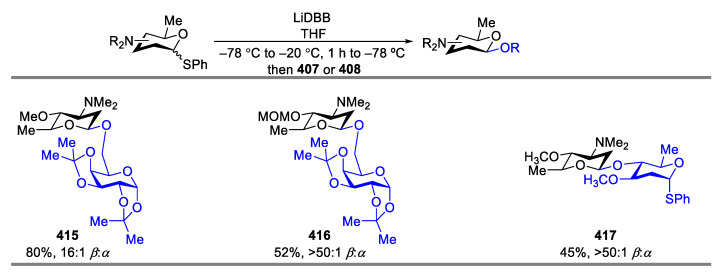
The synthesis of *β*-aminoglycoside.

Then, the synthesis of *β*-aminoglycoside was investigated (Scheme 52). The disaccharide **415** was obtained in 80% yield with satisfactory *β*-selectivity (*β*:*α* = 16:1) using THF as the only solvent. The disaccharide **417** was obtained in 45% yield with excellent *β*-selectivity (*β*:*α* > 50:1). Additionally, the methoxymethyl ether derivative **405** was transformed to the disaccharide **416** in 52% yield with excellent *β*-selectivity (*β*:*α* > 50:1) as well.

**Scheme 53:**
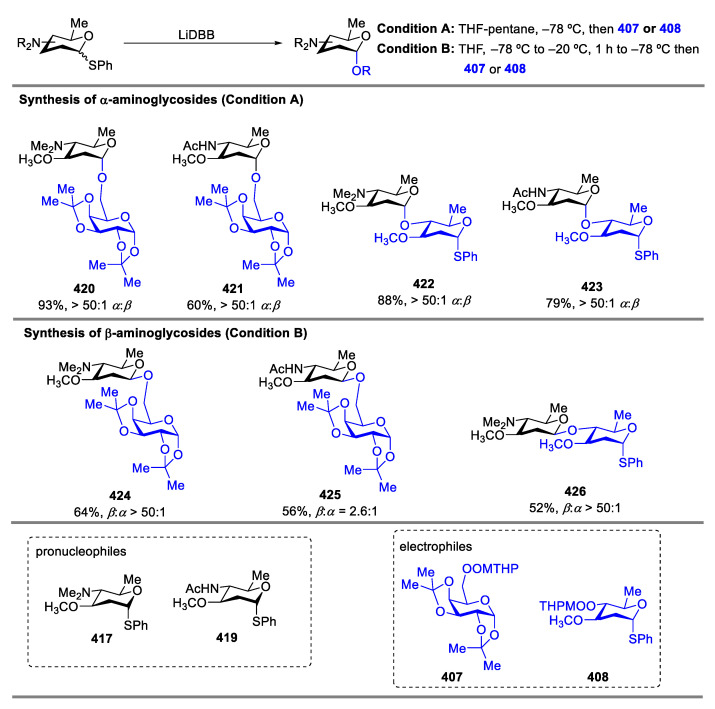
The synthesis of 4-aminoglycoside.

This methodology was also suitable for glycosylation of 4-amino-4-deoxysugars [116]. Coupling of acosamine derivatives **418** and **419** with MTHP electrophile **405** and **406** under Condition A generated desired disaccharides **420**–**423** in 60−93% yield with *α*-selectivity (Scheme 53). While disaccharide **424** was obtained in 64% yield with *β*-selectivity (*β*:*α* > 50:1) under Condition B (see Scheme 53 for detail). Under Condition B, disaccharide **426** was obtained in 52% yield with *β*-selectivity (*β*:*α* > 50:1) following addition of secondary electrophile **408**, and derivative **419** was transformed into disaccharide **424** in 56% yield with *β*-selectivity (*β*:*α* > 2.6:1).

**Scheme 54:**
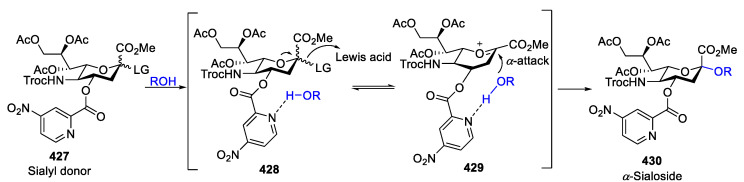
Design of potential sialyl donor for *α*-glycosylation.

Sialic acid (Neu5Ac) is an acidic nine carbon saccharide, which usually plays an essential role in terminating the carbohydrate portions of glycoproteins and glycolipids of mammals in the form of an *α*-glycoside. Silalylation via chemical methods with high *α*-stereoselectivity is challenging. For instance, the anomeric selectivity at the newly formed glycosidic bond is unpredictable due to the absence of a C3 participating group. On the other hand, the presence of the C1 electron-withdrawing carboxylic group could reduce the reactivity of sialic acid donor, resulting in a poor yield and formation of a significant amount of undesirable 2,3-glycal side-product. In view of the above challenges, Yang’s group developed an efficient *α*-sialylation methodology for various primary, secondary, and tertiary alcohol acceptors by using C4-Pico or 4-nitropicoloyl as directing group (Scheme 54) [166].

**Figure 7:**
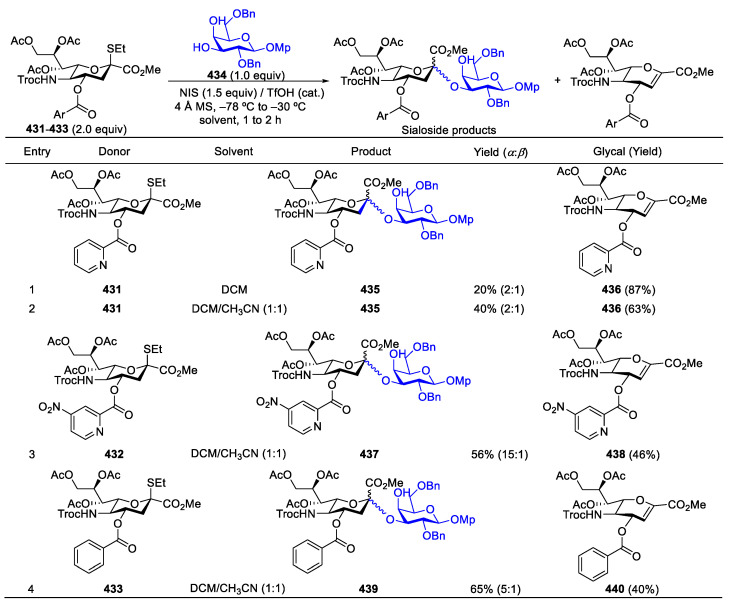
Glycosylation between donors **431**–**433** and acceptor **434**.

As for the scope of substrates, it was found that the combination of C4 Pico or 4-nitropicoloyl groups with the DCM/CH_3_CN [167,168] mixture solvent system is more effective and selective for *α*-glycosylation (Figure 7). Additionally, glycosylation of donor **432** with various acceptors all generated the desired products with satisfactory *α*-selectivity.

**Scheme 55:**
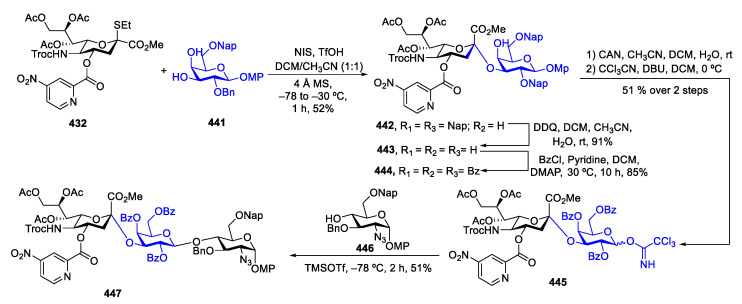
The synthesis of protected trisaccharide **447**.

In order to further explore this methodology, the synthesis of sialylated trisaccharide **446** was examined (Scheme 55). Coupling donor **432** with acceptor **441** provided sialylated disaccharide **442** in 52% yield with exclusive *α*-selectivity. Then, removal of the two 2-naphthylmethyl (Nap) protecting groups in **442** in the presence of 2,3-dichloro-5,6-dicyanobenzo-1,4-quinone (DDQ) gave the desired disaccharide triol **443** in 91% yield, which was followed by acylation with benzoyl chloride (BzCl) to produce **444** in 85% yield. Subsequently, deprotection of 1-*O*-*p*-methoxyphenyl group in **444** with ceric ammonium nitrate (CAN) followed by imidation with trichloroacetonitrile (CCl_3_CN) produced trichloroacetimidate **445** in 51% over two steps as inseparable *α*:*β* (1:1) isomers. Finally, coupling trichloroacetimidate **445** with monosaccharide alcohol **446** [169] under the activation of trimethylsilyl trifluoromethanesulfonate (TMSOTf) in DCM at −78 °C, generated the target trisaccharide **447** in 51% yield with exclusive *α*-selectivity.

Additionally, similar strategies of glycosylation with various picoloylated sialyl donors were reported by Cristina De Meo’s group [170,171] and other strategies are also reported [172,173,174,175]. There are also several elegant reviews about glycosylation of sialic acid [176,177,178]; we will not include these herein.

## 4. Conclusions

Amino sugars exist widely in nature and have a range of biological activities. The fine distinctions in their glycan structures may lead to the difference of the activities and, thus, can be exploited for the development of drugs and vaccines. The new methodologies and strategies for stereoselective aminoglycoside construction and convergent oligosaccharide assembly are critical for the development of carbohydrate chemistry. Many new leaving groups and catalyst systems have been developed with the goal to develop a versatile method for stereoselective glycosylation of amino sugars.

In this review, we summarized the advances of the stereoselective glycosylation protocols of amino sugars. A number of glycosylation strategies under mild conditions with potential applications mediated or catalyzed by transition-metal and (or) organic catalysts have been developed for stereoselective glycosylation of amino sugars. These methodologies render the glycosylation reaction more effective and extend the scope of donors and acceptors with a broad range of protecting groups, being no longer only dependent on neighboring group participation or anomeric effect. However, the results of these glycosylation methods are also affected by other factors, such as solvent effect, remote participation, steric hindrance, and conformational constraints, as well. After generous traditional approaches for the development of the synthesis of aminoglycosides, glycosidation of amino sugars via glycosyl radical intermediates has been a promising method and powerful tool, owing to its inherent features, including mild reaction conditions, broad functional groups tolerance, and being free of side reactions such as elimination and/or epimerization. Notably, the stereoselective formation of 1,2-*cis*-aminoglycosides via radical activation of glycosyl precursors is much less developed compared to conventional ionic activation strategies. In this context, it remains highly desirable to exploit general and user-friendly photocatalyzed or electrocatalyzed glycosylation methods to construct the challenging 1,2-*cis*-aminoglycosidic bonds that avoid sensitive reagents, strong (Lewis) acids, precious (metal) catalysts, or labile substrates.

## Data Availability

No new data were created.

## Abbreviations

Ac	acetyl
All	allyl
Ar	ary (substituted aromatic ring)
ABz	*o*-hexynylbenzoic acid
Bn	benzyl
Boc	*t*-butoxycarbonyl
BSM	benzenesulfinyl morpholine
BSP	1-benzensulfinylpiperidine
Bz	benzoyl
*n*Bu	*n*-butyl
*t*Bu	*t*-butyl
Cbz	benzyloxycarbonyl
DCM	dichloromethane
DNs	2,4-dinitrobenzenesulfonyl
Et	ethyl
Fmoc	9-fluorenylmethoxycarbonyl
HFIP	1,1,1,3,3,3-hexafluoro-2-propanol
KHMDS	potassium bis(trimethylsilyl)amide
Me	methyl
NIS	*N*-iodosuccinimide
Ns	*p*-nitrobenzene sulfonyl
Phth	phthaloyl
PMB (MP)	*p*-methoxybenzyl
*i*Pr	isopropyl
Ser	L-serine
TBAF	tetrabutylammonium fluoride
TBS	*t*-butyldimethylsilyl
TES	triethylsilane
Tf	trifluoromethanesulfony
Thr	L-threonine
TIPS	triisopropylsilyl
